# The influence of vegetation and soil characteristics on active‐layer thickness of permafrost soils in boreal forest

**DOI:** 10.1111/gcb.13248

**Published:** 2016-06-09

**Authors:** James P. Fisher, Cristian Estop‐Aragonés, Aaron Thierry, Dan J. Charman, Stephen A. Wolfe, Iain P. Hartley, Julian B. Murton, Mathew Williams, Gareth K. Phoenix

**Affiliations:** ^1^ Department of Animal and Plant SciencesUniversity of SheffieldWestern BankSheffieldS10 2TNUK; ^2^ GeographyCollege of Life and Environmental ScienceUniversity of ExeterExeterEX4 4RJUK; ^3^ School of GeoSciencesUniversity of EdinburghKings BuildingsEdinburghEH9 3JNUK; ^4^ Geological Survey of CanadaNatural Resources CanadaOttawaONK1A 0E8Canada; ^5^ Department of GeographyUniversity of SussexBrightonBN1 9QJUK

**Keywords:** active‐layer thickness, boreal forest, discontinuous zone, Northwest Territories, permafrost, structural equation modelling

## Abstract

Carbon release from thawing permafrost soils could significantly exacerbate global warming as the active‐layer deepens, exposing more carbon to decay. Plant community and soil properties provide a major control on this by influencing the maximum depth of thaw each summer (active‐layer thickness; ALT), but a quantitative understanding of the relative importance of plant and soil characteristics, and their interactions in determine ALTs, is currently lacking. To address this, we undertook an extensive survey of multiple vegetation and edaphic characteristics and ALTs across multiple plots in four field sites within boreal forest in the discontinuous permafrost zone (NWT, Canada). Our sites included mature black spruce, burned black spruce and paper birch, allowing us to determine vegetation and edaphic drivers that emerge as the most important and broadly applicable across these key vegetation and disturbance gradients, as well as providing insight into site‐specific differences. Across sites, the most important vegetation characteristics limiting thaw (shallower ALTs) were tree leaf area index (LAI), moss layer thickness and understory LAI in that order. Thicker soil organic layers also reduced ALTs, though were less influential than moss thickness. Surface moisture (0–6 cm) promoted increased ALTs, whereas deeper soil moisture (11–16 cm) acted to modify the impact of the vegetation, in particular increasing the importance of understory or tree canopy shading in reducing thaw. These direct and indirect effects of moisture indicate that future changes in precipitation and evapotranspiration may have large influences on ALTs. Our work also suggests that forest fires cause greater ALTs by simultaneously decreasing multiple ecosystem characteristics which otherwise protect permafrost. Given that vegetation and edaphic characteristics have such clear and large influences on ALTs, our data provide a key benchmark against which to evaluate process models used to predict future impacts of climate warming on permafrost degradation and subsequent feedback to climate.

## Introduction

The mass of the permafrost carbon (C) stock is estimated to be almost twice that of the atmosphere, totalling ca. 1300 Pg (Hugelius *et al*., 2014). As permafrost thaws an increasing amount of previously frozen C is exposed to microbial decomposition and hence can be transferred to the atmosphere and hydrosphere (Zimov *et al*., 2006; Schuur *et al*., 2009; Schaefer *et al*., 2011). This transfer is of major concern given that high latitudes are predicted to experience the fastest rate of warming compared to the rest of the globe (IPCC, 2013). Observations over recent decades demonstrate that permafrost is warming, thinning and shrinking in area (Romanovsky *et al*., 2010). Therefore, to accurately predict the release of carbon from thawing permafrost and its feedback to climate, it is essential to fully understand the controls on permafrost thaw.

In the early stages of permafrost degradation, thickening of the active layer (the seasonally thawed soil layer above permafrost in which biological activity takes place) is thought to be the dominant process (Schuur *et al*., 2008). Although climatic warming is important in increasing active‐layer thickness (ALT), the strength of the relationship between air temperature and ALT varies substantially between different regions and may be strongly influenced by factors such as vegetation cover and edaphic properties (Jorgenson *et al*., 2010; Shiklomanov *et al*., 2010; Shiklomanov & Nelson, 2013). As a result of the surface offset (the difference between air temperature and near‐surface ground temperature) provided by ground cover and surface conditions, permafrost can persist in areas where the mean annual air temperature (MAAT) is as high as +2 °C, or degrade in areas where MAAT is −20 °C (Jorgenson *et al*., 2010). Therefore, along latitudinal gradients, increasing vegetation cover southward may compensate for greater summer warmth, weakening the relationship between MAAT and ALT (Walker *et al*., 2003). At finer scales, within catchments or hill slopes, ecosystem characteristics may play the dominant role in driving ALT (Jorgenson *et al*., 2010).

Several vegetation characteristics can influence soil temperature and hence ALT. Increasing leaf area reduces the amount of radiation reaching the soil, which should act to reduce ALT and hence protect permafrost (Marsh *et al*., 2010). However, with increasing stem density, particularly in shrubby species, vegetation can trap more snow, which insulates the ground and reduces heat loss in winter, potentially increasing ALT in the subsequent thaw season (Sturm *et al*., 2001). Experimental removal of shrub or dwarf shrub and nontussock sedge cover in Siberian and Alaskan tundra has been shown to increase ALT considerably (Kade & Walker, 2008; Blok *et al.,* 2010). In the boreal region, the tree canopy leaf area performs a similar shading role to that of the understory, but evergreen canopies also trap snow aloft and reduce snow cover on the ground. This trapping may increase conductive heat loss from the ground in winter, which may decrease ALTs and so protect permafrost (Yi *et al*., 2007).

Mosses are another important component of high latitude vegetation (Street *et al*., 2012, 2013), and have been largely neglected in coupled C‐climate models (Turetsky *et al*., 2007, 2012). Mosses strongly dampen temperature fluctuations in the soil, largely because their open structure makes them effective insulators. However, their thermal conductivity is strongly influenced by their moisture content (Gornall *et al*., 2007; O'Donnell *et al*., 2009). In summer, a dry moss layer minimizes downward heat conduction, whereas when wet during the shoulder seasons, and when frozen in winter, the higher thermal conductivity increases upward heat conduction (Burn & Smith, 1988). Both processes keep the ground cool, thus reducing ALTs. Because the thermal properties of mosses can be explained solely by their physical properties (such as mat thickness and moisture content), this should simplify their inclusion in processed‐based models (Soudzilovskaia *et al*., 2013).

The thickness of the soil organic layer beneath the moss layer performs a similarly important role in determining ALT (Johnson *et al*., 2013). The low bulk density of organic relative to mineral soils means organic soils can present more varied and extreme air and water contents, leading to a much greater range of thermal conductivities and specific heat capacities. Moisture content plays a major modifying role in the thermal properties of the soil organic layer, as it does for moss (O'Donnell *et al*., 2009), and can also influence ALT by nonconductive heat transfer through movement of liquid water and vapour (Hinkel & Outcalt, 1994; Kane *et al*., 2001). ALT monitoring in Canada has revealed that within‐site variation is much reduced at sites with homogeneous thin organic layers, but where large variations in organic layer thickness or its water content exist, ALT is much more variable (Smith *et al*., 2009).

Our goal here is to better understand the magnitude of effects and relative importance of the multiple vegetation and edaphic characteristics that influence ALT. Such an understanding is particularly important given that climate change is likely to have contrasting impacts on different ecosystem characteristics. For instance, the tree line will (overall) move poleward with climate warming while tundra shrub cover is also predicted to increase (Grace, 2002; Jia *et al*., 2009; Forbes *et al*., 2010). Greater shrub cover, however, is likely to reduce moss cover and may over time result in thinner organic layers (Walker *et al*., 2006). Furthermore, fire activity in boreal forest and tundra is likely to increase in the future, causing further changes to vegetation structure and organic layer thickness and strongly influencing soil moisture (Stocks *et al*., 1998 Kelly *et al*., 2013). Additionally, these factors affecting ALT are likely to be of particular importance within the discontinuous and sporadic permafrost regions, which are typically dominated by boreal forests, as these areas have relatively warm (−0.2 °C) and thin permafrost, which may be particularly vulnerable to thaw (Smith *et al*., 2005; Baltzer *et al*., 2014).

Here, we aim to quantify the influence and importance of vegetation and soil characteristics in driving ALT in boreal forests, which cover over 50% of permafrost regions globally (Osterkamp *et al*., 2000). Our study includes four field sites within the discontinuous permafrost zone in the Northwest Territories, Canada. These incorporate different fire histories, substrates and tree canopies (deciduous or evergreen) that capture three representative and contrasting boreal forest cover types [black spruce (*Picea mariana*) at two sites of differing canopy density, a burned black spruce site and a paper birch (*Betula papyrifera*) site]. We employed a stratified sampling strategy to encompass the full range of variation in vegetation and edaphic characteristics within each site to produce the most detailed fine scale survey of the links between ALT, vegetation and soil characteristics to date. Specifically, we hypothesized that (i) increasing canopy and understory LAI would decrease ALT; (ii) taller understory vegetation would increase ALT, (iii) increasingly thick moss and soil organic layers would decrease ALT and (iv) increasing soil moisture would increase ALT. In addition to addressing these hypotheses, our approach allowed us to determine the relative importance of these different drivers of ALT, and how they interact to determine ALTs.

## Materials and methods

### Study sites

All study sites were located on gently sloping topography, avoiding low points in the landscape where permafrost may impede drainage and create wetlands (median slope angle about 6–8°; Table S1). Thus, we selected sites where the permafrost dynamics were likely to be controlled by ecosystem properties rather than conversely the permafrost controlling ecosystem properties (see Discussion).

**Figure 1:**
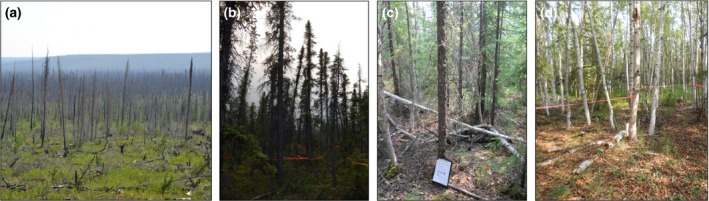
Field sites. (a) Mosquito Spruce Burned (MSB), (b) Mosquito Spruce Unburned, (c) Boundary Creek Spruce (BS) and (d) Boundary Creek Birch (BB). Field site locations shown on map Fig. S1.

**Figure 2:**
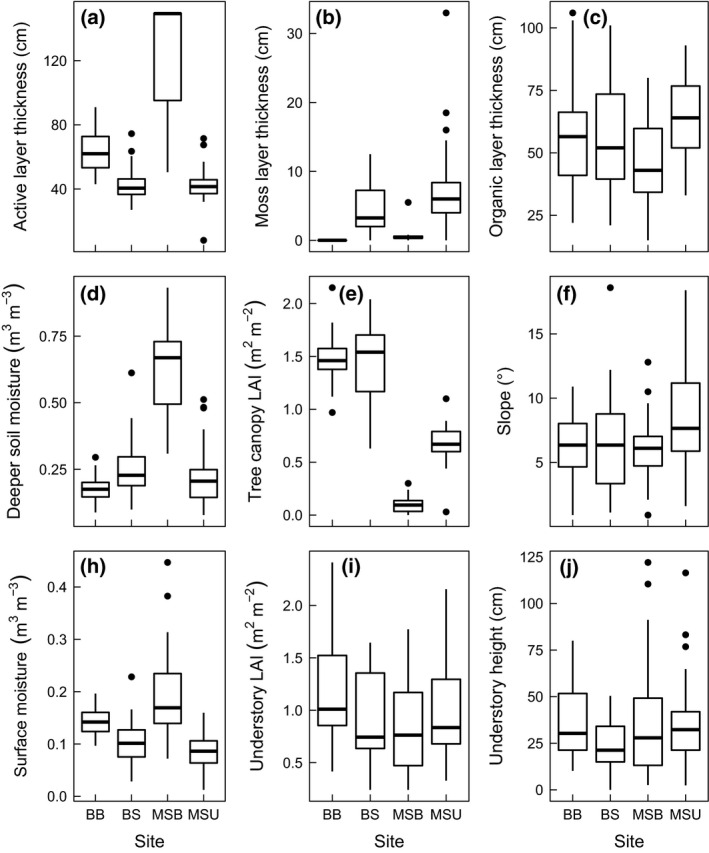
Boxplots of ALT and all vegetation and soil characteristics measured at each site. BB is Boundary Birch, BS is Boundary Spruce, MSB Mosquito Spruce Burned and MSU is Mosquito Spruce Unburned. Boxplots represent median, 1st and 3rd quartiles (line and box), whiskers represent maxima and minima and points represent outliers.

Two study sites in the Great Slave Lowland High Boreal Ecoregion (Fig. S1) were adjacent burned and unburned areas of black spruce (*Picea mariana*) forest located near Mosquito Creek, NWT (62°42′2.3″N, 116°8′8.8″W), subsequently referred to as Mosquito Spruce Burned (MSB) and Mosquito Spruce Unburned (MSU) (Fig. 1). The effects of a large fire at this site in 2008 (Canadian Forest Service, 2014) were still clearly visible in our survey year (2014). The burned site was characterized by charred snags and ground scorching, bare ground coverage and associated heterogeneous losses of moss and organic soil horizon thickness. In some areas, taller shrub birch (*Betula glandulosa*) had begun to establish, while other patches were covered with shrubby species such *as Rhododendron groenlandicum* and *Vaccinium vitis‐idaea* (Fig. S2). The density of dead trees was 2720 stems ha^−1^, with a mean diameter at breast height (DBH) of 6.8 ± 0.3 cm. A neighbouring (ca. 50 m) study site was established in an unburned area (ca. 500 m^2^ of unburned forest) dominated by mature black spruce trees interspersed with tamarack (*Larix laricina*), with a varying degree of canopy closure (stem density 4161 stems ha^−1^, DBH 7.8 ± 0.2 cm, Fig. 2, Table S1) and a carpet of feather mosses, predominantly *Hylocomium splendens*. Again, shrub and herbaceous species, mainly *Arctostaphylos rubra* and *Geocaulon lividum*, formed a patchy understory (Fig. S2). The soil profile at these sites transitioned sharply from organic to mineral soil, and the mineral horizon was generally poorly pore‐ice cemented and, down to 1 m depth, dominated by grey sand with occasional rounded to subangular pebbles <2 cm in diameter. Texturally, the <2 mm fraction of mineral soil at MSU had mean values (*n* = 6) of 82 ± 8% sand, 11 ± 6% silt and 7 ± 4% clay, similar to those at MSB (*n* = 6) of 79 ± 5% sand, 15 ± 4% silt and 6 ± 2% clay.

Two other sites were established ca. 63 km east in the Great Slave Lowland within adjacent black spruce and paper birch (*Betula papyrifera*) forest near Boundary Creek (62°31′36.3″N, 114°57′41.3″W and 62°31′37.7″N, 114°57′38.9″W respectively), subsequently referred to as Boundary Creek Spruce (BS) and Boundary Creek Birch (BB) (Figs 1 and S1). Paper birch stem density was 2980 stems ha^−1^ and mean DBH was 9.6 ± 0.3 cm (Fig. 2 and Table S1 for LAI_Tree_). There was no moss layer in the birch understory; instead the ground was covered with leaf litter with sparse patches of fireweed (*Chamerion angustifolium*), low stature shrubs such as *Ribes glandulosum* and *Rubus chamaemorus* or denser stands of *Rosa acicularis* or emergent *P. mariana* saplings (Fig. S2). Nearby, the spruce site (BS) had a stem density of 6620 stems ha^−1^ and the trees had a mean DBH of 5.7 ± 0.1 cm. The understory was similar to that of the Mosquito Creek unburned site though with some fruticose lichen dominated patches. The organic soil horizon at these sites was underlain, to at least 1 m depth, by grey silty clay mineral soil with ice lenses <1 cm thick and disseminated ice crystals a few millimetres in diameter. Texturally, the <2 mm fraction of mineral soil at BS had mean values (*n* = 6) of 13 ± 11% sand, 24 ± 5% silt and 63 ± 15% clay, and at BB (*n* = 4) 26 ± 10% sand, 29 ± 10% silt and 45 ± 20% clay. Abundant segregated ice was commonly observed in the BS soil profiles at depths between 60 and 90 cm, which coincides with the top of the permafrost.

During the growing season, the average mean daily air temperature at both the Mosquito Creek and Boundary Creek sites, measured using screened TinyTag probes (Gemini, Chichester, UK), was 15.6 °C, the average daily maximum and minimum temperatures were 21.6 and 8.2 °C respectively (Fig. S3a). Total rainfall through the growing season, measured at the nearby Environment Canada Yellowknife‐Henderson station (62°27′00.0″N, 114°22′48.0″W) was 77 mm (Fig. S3b). At all sites, bottom‐sealed access tubes filled with antifreeze were installed after soil coring to monitor soil temperature profiles by means of sealed thermistors connected to a digital multimetre. Soil temperatures, at 1 m depth obtained from access tubes installed after soil coring, at the end of the growing season (29 August 2014) were −0.2 ± 0.2 °C in MSU and 1.6 ± 1.6 °C in MSB. Soil temperatures at 1 m depth at the end of the growing season (2nd September 2014) were −0.5 ± 0.1 °C in BS and −0.4 ± 0.1 °C in BB.

### Plot establishment

In 2014, plots of 1 m^2^ were located to ensure that a full range of ground cover types, tree canopy cover and moss and organic layer thicknesses were sampled at each site. Additionally, we only selected plots where the ground cover was homogeneous over an area of at least 2 × 2 m such that the 1 m^2^ study plot was representative of the larger area. Initially, 30 plots were established at each of the four study sites. However, as the summer thaw season progressed it became apparent that, for a small number of these plots, thaw depth could not be accurately determined due to the resistance of unfrozen, clay‐rich soils or rocks. Hence the final dataset had 30 plots from the MSB site, 30 from the MSU site, 24 from the BS site and 24 from the BB site, giving a total of 108 plots.

### Vegetation and edaphic characteristics

Vegetation characteristic survey work was carried out between 27th July 2014 and 25th August 2014, with thaw depths recorded on 28th August – 1st September 2014 to capture near maximum ALT.

Tree canopy leaf area index (LAI_Tree_) was determined using a Nikon D5000 DSLR camera with a Sigma EX 4.5 1:2.8 DC HSM hemispherical lens. Nine photographs were taken at different locations 1 m above each plot and processed with CAN‐EYE software (Weiss & Baret, 2010). The ‘LAI2000, 5 rings.Eff’ output was used for maximum comparability with understory LAI. To control for influence of sunlight conditions, images were thresholded individually by the same researcher, allowing the threshold to be set appropriately for the sky conditions. Whenever possible all LAI measurements were taken as late as possible in the day to reduce influence of directly incident sunlight.

Understory LAI (LAI_U_) was measured using a LI‐COR LAI2000 Plant Canopy Analyser (LI‐COR, Lincoln, USA). One measurement was taken above the understory canopy with a 90° field of view cap and four measurements were taken below the understory canopy at the four corners of a 20 × 20 cm square at the centre of each plot. This protocol allowed LAI_U_ to be determined independently of LAI_Tree_ (White *et al*., 1997). LAI_U_ therefore comprises the vascular plant component but not the moss ground layer. To control for sunlight conditions, the LAI2000 has a relatively narrow (90°) field of view cap that minimizes the impact of uneven cloud conditions on days when the sky was cloudy or nonuniformly overcast (as recommended in the user manual). When the sky was clear a tarpaulin was used to cast shade on the part of the understory canopy that was being measured (again following the manual).

The maximum understory vegetation height was measured from the moss or soil surface (where moss was not present) at the four corners and in the centre of a 50 × 50 cm quadrat in the centre of the 1 m^2^ plot. The mean of these five values was used in subsequent analyses.

Moss thickness was determined by carefully removing a section of moss and organic material from the ground with a serrated knife while avoiding compression. Moss thickness was measured as the distance from the surface of the living moss to the point at which dead moss (fibric material) became decomposed to a state that its structure was no longer discernible. Moss thickness therefore includes living and dead moss layers.

Surface moisture was measured in the upper 6 cm of the moss/soil layer using an ML3 ThetaKit (Delta‐T Devices Ltd, Cambridge, UK) with an accuracy of 1% for 0–50% range volumetric moisture content, and precision of 1 mV. Measurements were taken by placing the probe gently into the surface of the moss carpet or soil surface (in the case of bare ground plots), and in most cases readings reflected the moisture in the moss layer. Given the similar properties of soil organic matter and moss in insulating permafrost, we deemed it more appropriate to measure the depth of the moisture measurement from the surface of the soil or moss when present, rather than always measuring from the soil surface (even when there was a moss layer above this). A deeper soil moisture measurement was taken by parting the moss/organic layer so that the probe was inserted to a depth of about 11 cm, hence the measurement volume was between ca. 11–16 cm below the surface of the moss or soil. For clarity, we refer to this reading as deeper soil moisture, though in the thickest moss areas, this 11–16 cm volume included some moss. Four measurements were taken at each plot throughout the growing season and the mean of these readings was used for data analysis. In previous work, we found that millivolt readings varied over small scales even within the same plot, so building new calibration curves for the moisture probe was problematic (and ideally would have needed a calibration curve for each plot). Hence, we used the standard factory calibration settings for organic soils and concentrated effort in obtaining multiple readings from each plot at the multiple sites over the growing season.

Soil organic matter (OM) thickness, determined using a soil corer (1.8 cm internal diam.), was measured to the depth of the base of the O horizon. The thickness of the moss layer was then subtracted from this measurement. Slope was measured, as it influences surface water runoff, snowpack depth and solar radiation interception. A 50 cm wooden plank was laid along the steepest gradient through the centre of each plot, and the angle below the horizon was measured using a digital angle meter with a bubble level.

ALT was measured using a graduated stainless steel rod (1.5 cm diameter) inserted to the point at which it was impeded by frozen soil (Nelson & Hinkel, 2003). At this late stage in summer, thaw depth approaches its maximum and is therefore close to that of the ALT (Walker *et al*., 2003). A temperature probe was used to confirm that the soil was frozen (0 °C) at the point of refusal. The probe was custom built (British Rototherm Co. Ltd. Port Talbot, UK), and consisted of a robust 1.3 m long tube of stainless steel (11 mm outer diameter, 7 mm inner diameter) fitted with a 300 mm wide ‘T’ handle for inserting and extracting the probe from the soil. The sensing tip was 7 mm in outer diameter, sharpened to a point and contained a platinum resistor (100 Ω at 0 °C, 4 wire, Class B, made to IEC 751 Standard; manufacturer's stated tolerance ±0.3 °C). Temperature measurements were made by connecting the probe to a hand‐held digital thermometer. Where temperatures were >0 °C, these plots were excluded from analysis.

### Statistical analyses

The influence of the measured vegetation and edaphic factors on thaw depth was assessed using both tobit multiple regression models and structural equation modelling (SEM). These approaches are complementary, with SEM giving a more mechanistic insight into the controls on ALT whereas multiple regression modelling provides a clearer insight into the direct influence of the measured variables on ALT. In addition to their complementary strengths, the combined approach reinforces confidence in the conclusions drawn where there is agreement between them.

Tobit regression was used in place of standard multiple regression because 17 of the plots in the burned sites had ALTs greater than 150 cm, which were beyond the limit of the probe, resulting in a censored response variable. Tobit models were developed specifically to deal with this kind of censored data (Tobin, 1958) and were implemented in the *VGAM* R package (Yee, 2014). Prior to tobit analysis predictor variables were mean centred to aid interpretation of the results. Curvature in the relationship between explanatory and response variables was tested by fitting all explanatory variables and their squared terms then retaining only those terms which were significant in the full model. A series of models each containing main effects and a subset of all possible two‐way interactions were used to identify potentially significant interactions. A full model was then constructed using all main effect terms plus the identified potentially significant interactions and quadratic terms. This model was simplified by sequentially removing nonsignificant terms in order to obtain the model with minimum Akaike information criterion (AIC) (Crawley, 2012). *α* levels for testing the significance of the terms remaining in the model were determined using the false discovery rate control method described by Benjamini & Hochberg (1995). Interactions were interpreted using the methods of Aiken & West (1991). Model fits were checked visually to ensure that they conformed to model assumptions.

This process was repeated at the individual site level to determine whether the same factors that emerged as important drivers of ALT across all sites were also significant within each land cover type (paper birch, black spruce and burned black spruce). However, interaction terms were not fitted as the sample size within each land cover type was too small, and further data collection within each individual site to the level needed to elucidate interactions would have been impossible in the time available. Data are back transformed for presentation in figures to ease visualization of relationships, but our interpretations are based on the nonback transformed data in the statistical analysis. These analyses were carried out using R 3.1.2 (R Core Team, 2014).

The data were also interrogated with SEM, which allows links between measured variables to be analysed with direct paths implying causality and indirect paths occurring where the impact of one factor is modified by another. Using SEM, direct and indirect impacts of exogenous and endogenous variables can be estimated and compared. Additionally SEM is well suited to data where there may be colinearity among predictor variables, since SEM can be used to build meaningful models of ecological systems where this is present (Graham, 2003). Bayesian SEM was used as it allowed the incorporation of the censored ALT measurement (burned site plots with ALT >150 cm) by restricting the posterior distribution of those ALT estimates which could not be measured directly. Diffuse priors were set for all parameter estimates except where an admissibility test determined that the lower bound of the prior needed to be set to zero in order to generate a proper solution. The rationale behind the structure of the model is outlined in Supporting Information. Ninety‐five percentage highest density intervals (HDIs) were used to assess whether parameter values and differences between parameter values were credibly different from zero (i.e. zero did not lie within the 95% HDI). SEM was carried out using IBM SPSS Amos 22 (Arbuckle, 2013).

## Results

### Site characteristics

Site characteristics contrasted as expected for these land cover units. Briefly, MSU had relatively low average surface and deep soil moisture contents, an intermediate tree canopy LAI, and thick moss (dead + live moss) and organic layers (Fig. 2, Table S1). BS was similar, but had a more closed tree canopy, slightly greater deep soil moisture, and slightly lower moss and OM thicknesses (Fig. 2, Table S1). MSB had the greatest surface and deep soil moisture content, lowest tree canopy LAI, OM thickness, and a very thin moss layer (Fig. 2; Table S1). The BB site almost completely lacked moss, instead having a layer of leaf litter, but the total organic layer thickness was similar to that of the BS site (Fig. 2b, c; Table S1). Surface moisture was intermediate at this site, but deep soil moisture content was greater than in the neighbouring BS site (Fig. 2d, h; Table S1). Tree canopy LAI was very similar across both of the Boundary Creek sites. ALTs were greatest at the MSB site, intermediate at BB and smallest at both MSU and BS (Fig. 2a, Table S1).

### Cross‐site tobit multiple regression analysis

**Figure 3:**
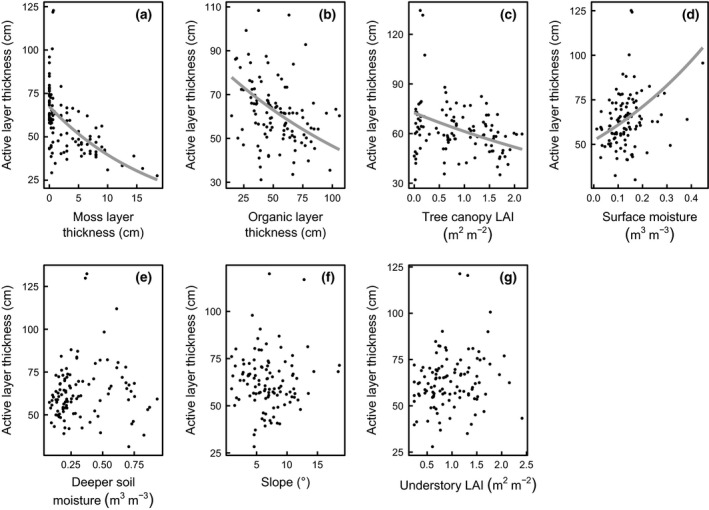
Partial residual plots for the main effects in the multiple regression model. Points represent active‐layer thickness (ALT) when all factors are held at their median values (partial residuals) and regression lines are derived from the multivariate tobit regression model. Partial residuals and regression lines (only presented where a significant main effect was found in the tobit model) have been back transformed to the original scale of ALT (exponential transformation with the base *e*).

**Figure 4:**
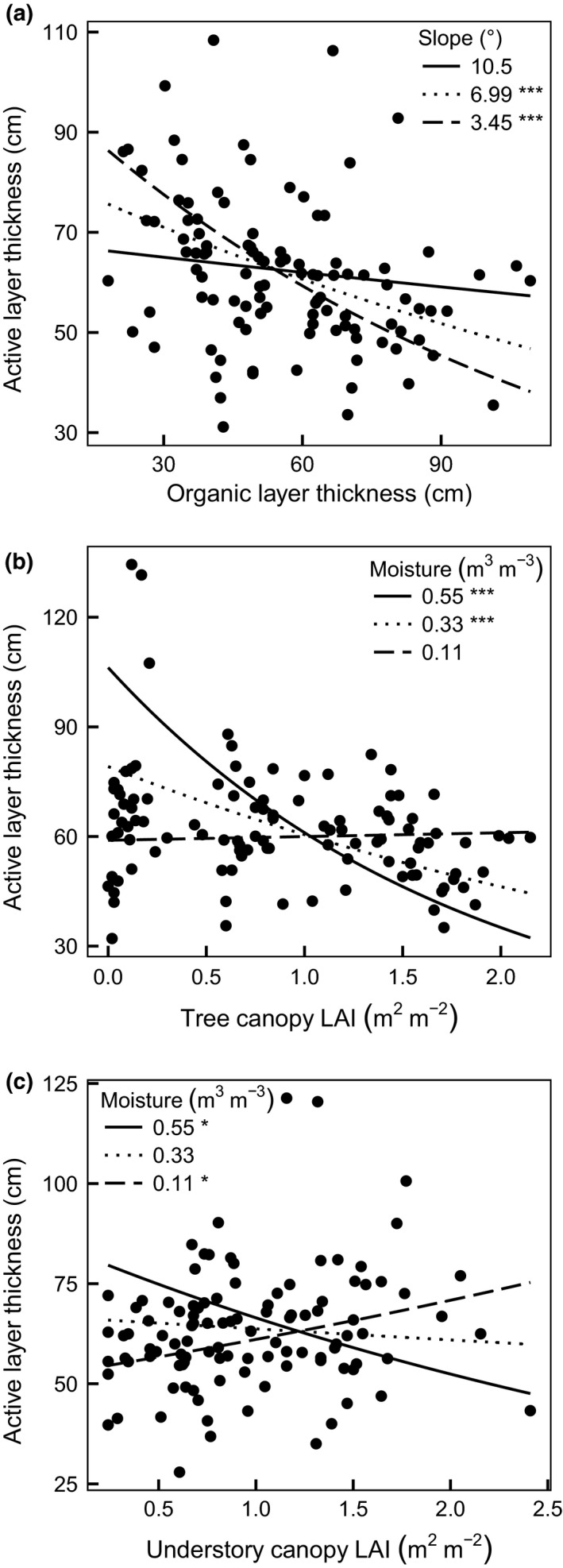
Interaction plots with partial residuals derived from the multiple regression model. (a) Interaction between organic layer thickness (OLT) and slope; points represent partial residuals for OLT; dotted, solid and dashed lines show the relationship between active‐layer thickness (ALT) and OLT when slope is at its mean value (6.99°), one standard deviation (SD) above its mean value (10.5°) and one SD below its mean value (3.45°) respectively. (b) Interaction between tree canopy LAI (LAI_T__ree_) and deeper soil moisture (11–16 cm depth); points represent partial residuals for LAI_T__ree_; dotted, solid and dashed lines show the relationship between ALT and LAI_T__ree_ when deeper soil moisture is at its mean value (0.33 m^3^ m^−3^), one SD above its mean value (0.55 m^3^ m^−3^) and one SD below its mean value (0.11 m^3^ m^−3^) respectively and (c) Interaction between understory canopy LAI (LAI_U__nderstory_) and deeper soil moisture; points represent partial residuals for LAI_U__nderstory_; dotted, solid and dashed lines show the relationship between ALT and LAI_U__nderstory_ when deeper soil moisture is at its mean value (0.33 m^3^ m^−3^), one SD above its mean value and one SD below its mean value (0.11 m^3^ m^−3^) respectively. Significant stars in figure legends for individual relationships are as follows (*P <* *0.05, **P <* *0.01, ***P <* *0.001).

The tobit multiple regression of all site data combined revealed significant effects of OM thickness, moss layer thickness, surface moisture and LAI_Tree_ on ALT (detailed below, Table 1, Fig. 3). ALT was also influenced by significant interactions between LAI_Tree_ and deeper soil moisture, between LAI_U_ and deeper soil moisture and between OM thickness and ground slope angle (Table 1, Fig. 4). The combination of factors retained in the final model (OM thickness, moss layer thickness, LAI_U_, LAI_Tree_, slope, deeper soil moisture, surface moisture and the interactions detailed in Table 1) explained 73% of the variation in ALT across our four sites (Adjusted McFadden's pseudo *R* ^2^ = 0.734).

**Table 1:** Parameter estimates for a tobit regression model of the effect of the vegetation and edaphic variables on log(active‐layer thickness); *n *=* *108, Adjusted McFadden's *R* ^2^ = 0.734. Parameters in bold are significant at the *α *= 0.0375 level (*α* determined using false discovery rate control method). Analysis is for all sites combined. Units shown in square brackets are those used before log transformation

	Parameter estimate	SE	*t*	*P*
(Intercept) [cm]	**4.043**	**0.038**	**105.599**	**<0.001**
OM thickness [cm]	**−0.005**	**0.001**	**−3.844**	**<0.001**
Moss thickness [cm]	**−0.053**	**0.011**	**−4.936**	**<0.001**
Understory LAI [m^2^ m^−2^]	−0.044	0.056	−0.799	0.424
Tree Canopy LAI [m^2^ m^−2^]	**−0.268**	**0.06**	**−4.449**	**<0.001**
Slope [°]	0.002	0.008	0.256	0.798
Deeper moisture [m^3^ m^−3^]	0.21	0.238	0.885	0.376
Surface moisture [m^3^ m^−3^]	**1.549**	**0.574**	**2.698**	**0.007**
(Deeper moisture)^2^	−1.495	0.837	−1.785	0.074
Moss thickness*Deeper moisture	−0.095	0.054	−1.748	0.08
OM thickness*Tree LAI	0.005	0.002	2.139	0.032
OM thickness*Slope	**0.001**	**0.000**	**2.938**	**0.003**
Understory LAI*Deeper moisture	**−0.882**	**0.289**	**−3.054**	**0.002**
Tree LAI*Deeper moisture	**−1.305**	**0.448**	**−2.912**	**0.004**

As moss layer thickness increased ALT decreased. This relationship took the form of an exponential decay, suggesting that increases in shallow moss layers had a greater impact on ALT than in deeper moss layers (Fig. 3a, Table 1).

Similarly, increasing OM thickness decreased ALT overall (Fig. 3b). However, there was also an interaction between OM thickness and slope (Table 1, Fig. 4a). This interaction arose from a decreasing influence of increasing organic layer thickness on ALT with steeper slopes. For plots on the steepest slopes, increasing OM thickness only weakly reduced ALT (Fig. 4a).

The relationship between surface moisture and ALT was straightforward, with greater surface moisture resulting in greater ALTs (Table 1, Fig. 3d). In contrast, deeper soil moisture influenced ALT by modifying the influence of other factors (interactions described below).

Overall, increasing LAI_Tree_ caused a decrease in ALT (Table 1, Fig. 3c). This effect, however, was moderated by a significant interaction with deeper soil moisture (Table 1, Fig. 4b). As deeper soil moisture declined, the influence of LAI_Tree_ on ALT was also decreased. Specifically, when soil moisture was high (one standard deviation above the mean, 0.55 m^3^ m^−3^) the relationship between LAI_Tree_ and ALT was strongly negative (Fig. 4b), but became less negative when soil moisture was at its mean (0.33 m^3^ m^−3^). The relationship between LAI_Tree_ and ALT was not significant when soil moisture was low (one standard deviation below the mean, 0.11 m^3^ m^−3^; Fig. 4b).

ALT was also influenced by a similar interaction between LAI_U_ and deeper soil moisture (Table 1). At the mean value of deeper soil moisture (0.33 m^3^ m^−3^), increasing LAI_U_ did not have a significant impact on ALT (Fig. 4c). However, at one standard deviation above the mean soil moisture (0.55 m^3^ m^−3^), increasing LAI_U_ decreased ALT, while at one standard deviation below (0.11 m^3^ m^−3^) increasing LAI_U_ increased ALT (Fig 4c).

### Individual land cover type tobit regression analysis

Different combinations of vegetation and edaphic factors were revealed to be the most important determinants of ALT within each land cover type (for brevity, greater detail for the site‐specific analyses, and discussion, are provided in supporting information, Table S2). Across the two black spruce sites (MSU and BS), increasing OM thickness and increasing LAI_Tree_ both resulted in decreasing ALTs. Conversely, increasing surface moisture content promoted greater ALTs (Table S2).

At the paper birch site (BB), only LAI_Tree_ had a significant effect on ALT, with more closed tree canopies associated with smaller ALTs (Table S2). In the burned black spruce site (MSB), LAI_U_ was the only significant factor, with greater LAI_U_ resulting in smaller ALTs (Table S2).

### Structural equation model

**Figure 5:**
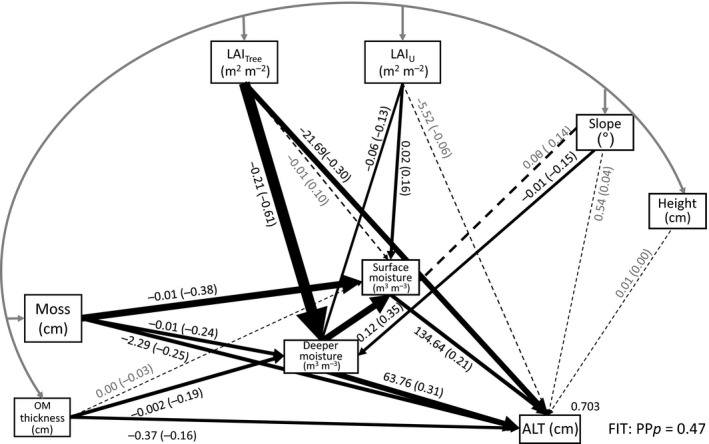
Results of Bayesian structural equation model assessing the direct and indirect (soil moisture mediated) impact of vegetation and edaphic characteristics on ALT. Solid lines represent paths where the 95% highest density interval (HDI) for the coefficient did not include zero, whereas dashed lines included zero in the 95% HDI. The unstandardized path coefficient is shown on each path with the standardized coefficient in parentheses, with line thicknesses scaled in proportion to their standardized path coefficient. The curved grey arrow represents the covariance between the exogenous variables which is not displayed here to aid presentation. The overall posterior predictive *P* value for the model is 0.46 (with values close to 0.5 indicating close agreement with the data) and the model explained 70.5% of the variance in ALT. Convergence was achieved after 5907 iterations (convergence statistic <1.002).

The structural equation model supported our *a priori* interpretation of the importance of moisture mediation on the impact of the vegetation and edaphic factors measured, and explained 70.5% of the variance in ALT (Fig. 5, Table S3, and supporting information for SEM rationale).

The effects observed are causal relationships in the context of the SEM approach where the choice of model ‐and so the direction of causality‐ is determined from the modeller's existing understanding of the system. Deeper soil moisture, LAI_Tree,_, moss layer thickness, surface moisture and OM thickness all had direct effects on ALT (95% highest density interval, HDI), and the standardized path coefficients indicated that the importance of these direct effects was in this order (Fig. 5). Increasing LAI_Tree_, moss layer thickness and OM thickness all had negative direct effects on ALT, whereas increasing deeper soil moisture and surface moisture both acted to increase ALT (95% HDI, Fig. 5).

LAI_Tree_, moss layer thickness, OM thickness and LAI_U_ had indirect effects on ALT, which were mediated via either surface or deeper soil moisture (zero not within 95% HDI for indirect effects). In the case of moss layer thickness, OM thickness and LAI_Tree_, the indirect effects via moisture acted to reinforce the direct effects. The indirect effects of LAI_U_ as mediated by moisture were more complicated as LAI_U_ simultaneously increased surface moisture and decreased deeper soil moisture. Moss layer thickness had a negative effect on both surface moisture and deeper soil moisture (95% HDI). LAI_Tree_ and OM thickness each had a negative effect on deeper soil moisture, but not on surface moisture, and LAI_U_ had a positive effect on surface moisture but not deeper soil moisture (zero not within 95% HDI). There was also a positive effect of deeper moisture on surface moisture. In the case of LAI_Tree_, moss layer thickness and OM thickness, the standardized direct effect of these variables was not credibly different to their standardized indirect (moisture mediated) effects (95% HDI of difference contains zero), implying that the indirect effect of these factors on ALT via soil moisture was of similar importance to their direct effects. However, the direct effect of deeper soil moisture on ALT was greater than the indirect effect via surface moisture.

## Discussion

This study provides the most comprehensive assessment of how ALT varies with vegetation and soil characteristics in boreal forest, and is the first to separate data on moss, vascular vegetation and soil organic layer properties. Our main approach of seeking emergent drivers that operate irrespective of land cover type shows that moss layer thickness and LAI_Tree_, in combination with soil OM thickness, are the most important and broadly applicable factors influencing ALT. However, the influence of both LAI_Tree_ and LAI_U_ on ALT is highly dependent on soil moisture, which also has its own direct effect of increasing ALTs. Therefore, the important central role that moisture plays in influencing ALT should not be underestimated given future changes in precipitation regimes and evapotranspiration that may significantly alter soil moisture. Indeed, all measured variables that had a direct impact on ALT also had indirect effects that were mediated by soil moisture. Understanding the direct effects and interactions of these key vegetation and soil characteristics is crucial both for understanding the controls on current ALTs, and also future impacts of climate change on permafrost degradation due to climate‐driven changes in vegetation and soil properties.

### Direct and indirect effects of soil moisture

Our SEM showed that surface soil moisture had the greatest direct impact on ALT, and also highlighted deeper soil moisture content as an important factor influencing ALT indirectly by modifying the effect of other factors. This is supported by the regression analysis that also revealed a significant influence of surface moisture in increasing ALTs, whereas the importance of deeper soil moisture lay in modifying the impact of other factors on ALT. Taken together, these findings strongly indicate that (among the factors we measured), future changes in precipitation and evapotranspiration patterns and hence associated changes in soil moisture, will have a particularly strong influence on the rate of permafrost degradation, both directly by increasing ALTs and indirectly by modifying the influence of other drivers (Iijima *et al*., 2010).

In the ecosystems we studied, the critical role of soil moisture emphasizes the importance of moss and organic matter layers as critical insulators, and the role of greater wetness in increasing heat conductance and resulting in deeper active layers. However, we note that in very wet and waterlogged soils in other systems, it is possible that greater moisture can alter ALTs due to the greater latent heat effect delaying freezing (Morse *et al*., 2015). Similarly, for wetland ecosystems where the water table is at or near the surface during the growing season, we expect changes in precipitation regime to have less effect on ALTs. Clearly, the critical role of soil moisture warrants further attention, for both empirical research and for model development.

### Moss layer thickness

Both regression analysis and SEM revealed that ALTs decreased with increasing moss layer thickness (live and dead moss). This is consistent with dry moss being a poor thermal conductor which readily insulates permafrost from warm summer air temperatures (Turetsky *et al*., 2012). The exponential nature of the regression relationship shows that while initial increments in moss layer thickness may have a large impact in reducing ALT, subsequent increases are less effective as has been noted in 1‐d modelling analysis (Riseborough *et al*., 2013). The SEM demonstrated that moss also affects ALT indirectly because increases in moss layer thickness resulted in drier soil. The tendency for the whole organic layer to dry in feathermoss covered soils has been demonstrated elsewhere (Harden *et al*., 1997) and a thicker moss layer may dry more quickly during hot, dry summers because its pore fraction is likely to be greater than that of the underlying OM. Overall, moss is the single most important component of the plant community in driving shallow ALTs and hence protecting permafrost.

### Organic matter thickness

Consistent with our hypotheses, thicker soil organic matter layers decreased ALTs, demonstrating the capacity of organic soils to protect permafrost from thaw by performing a similar role to that of the moss layer, with good insulating properties in summer when dry (O'Donnell *et al*., 2009). However, the actual relationship between OM thickness and ALT was more complicated due to a significant interaction with slope. Moisture may provide a mechanistic explanation for the OM thickness interaction with slope, where the influence of increasing OM thickness diminished on increasingly steep terrain. Because runoff and drainage can be improved on steeper terrain, as supported by our SEM analysis, the importance of increases in OM thickness is likely to be reduced because on a steeper, drier, slope less OM would be required to provide the same level of insulation compared to a shallower, wetter slope (Jorgenson *et al*., 2010). Steeper slopes may also have decreased snow pack thickness, reducing insulation from this and so furthering the importance of OM thickness on ALT (Nowinski *et al*., 2010; Johansson *et al*., 2013). This is consistent with OM thickness having a negative effect on deeper soil moisture in the SEM. The mechanism driving this is unclear but in thicker organic layers, the soil moisture reading at 11–16 cm may be situated within organic matter that is more ‘surface‐like’ and so less compacted and more freely draining (the opposite direction of influence of drier soils creating deeper organic layers seems very unlikely).

While the soil organic layer plays a similar role to the moss layer in insulating permafrost, a greater OM thickness may not compensate for a thinner moss layer; for instance a moss layer loss (e.g. from 15 to 0 cm) increases ALT by ca. 40 cm on average, whereas the same loss of OM thickness only increases ALTs by ca. 10 cm. Moss has a lower bulk density than the organic layer which will contribute to it draining/drying more readily in summer and having greater insulating properties (see also ‘justification for SEM design’ in supporting information). Additionally, although we did not measure the influence of litter, given the consistent emergence of moss as one of the most important factors influencing ALT, where moss is replaced by litter as the surface cover (as in deciduous vs. evergreen stands) it is highly unlikely that litter could maintain ALTs and hence protect permafrost to the extent that moss can.

### Leaf area index

Overall, increasing LAI_Tree_ caused a decrease in ALT, emphasizing the importance of a dense tree canopy in boreal forest in protecting permafrost. Three potential mechanisms could explain this observation; (i) increasingly closed evergreen canopies could intercept more snow, reducing the insulating snow pack and hence allowing more heat loss from the ground in winter (Lundberg & Koivusalo, 2003); (ii) larger tree canopies would transpire more, drying the soil and reducing thermal conductivity in summer, or (iii) a greater canopy would shade the ground more, reducing downward heat flux, as identified beneath shrubs in Siberian tundra (Blok *et al*., Heijmans, 2010). Our study suggests that the transpiration and shading mechanisms are both important in explaining the impact of LAI_Tree_ on ALT. Furthermore, whereas evergreen black spruce canopies at our study sites may intercept snow in winter, deciduous paper birch leafless canopies trap snow much less effectively, yet LAI_Tree_ still emerged as a significant factor for the birch site when analysed separately. Also, snow pack thickness, which typically exceeds 30 cm depth across this area has been suggested to be functionally homogeneous, and hence of limited importance in determining ALT (Morse *et al*., 2015). Nevertheless, moisture plays an important role in modifying LAI_Tree_ influence: as deeper soil moisture decreased, the strength of the impact of tree canopy shading also decreased because drier deeper soil will conduct less heat downwards, rendering the cooling effect of shading less important.

A similar interaction was also present between LAI_U_ and deeper soil moisture. However, in this case increasing LAI_U_ only decreases ALT in wetter soils. In drier soils, increasing LAI_U_ increases ALT, an effect which is not easy to explain, but could result from the prevention of further evaporation of moisture in these drier soils or from increased snow trapping. Also, LAI_U_ simultaneously increased surface moisture and decreased deeper soil moisture. That direction of causality (i.e. LAI_U_ driving moisture) in the SEM is likely for deeper soil moisture since greater LAI_U_ would dry soil more through greater transpiration, whereas the opposite direction of influence of more LAI_U_ occurring in drier soils is harder to accept. The mechanism for greater LAI_U_ increasing surface moisture is unclear, though greater shading and wind shelter may reduce surface evaporation from moss and litter layers.

Overall, LAI_U_ may have a smaller impact on ALT than LAI_Tree_ if they both influence ALT through shading. LAI_U_ may be less important under more closed tree canopies, such as those in the unburned black spruce and paper birch sites because any further shading they provide will have a lesser impact on the amount of radiation reaching the ground. However in the burned site, where the soil moisture content is greater and the tree canopy is absent, the vascular understory vegetation plays an important role in preventing greater active‐layer thickening. The rate of recovery of the ground layer vegetation postfire and prior to re‐establishment of trees could therefore be important in determining the extent of ALT deepening from fire, both through its shading effect and its capacity to dry the soil (Viereck *et al*., 2008).

### Fire impacts

The burned spruce site (MSB) had the greatest mean ALT of all our sites, consistent with it having the wettest soil, a very thin moss layer, and the thinnest organic layer – all factors promoting a deeper ALT. Indeed, with most of the other factors that control ALT reduced or removed by fire (OM thickness, moss depth, LAI_Tree_), only LAI_U_ remains as the main controlling factor in ALT across the burned site (Table S2). The interaction between LAI_Tree_ and soil moisture probably explains a large proportion of the considerable difference in ALT between our burned and unburned sites. The loss of the tree canopy postfire will increase soil moisture due to a lack of transpiration and may also allow the accumulation of more snow in winter. This is coupled with an increase in solar radiation reaching the ground, and a loss of insulation provided by reduced moss and organic layers, which will result in greater downward heat flux in summer and therefore greater ALT. Boreal forest fires are known to impact dramatically on ALT (Mackay, 1995; Burn, 1998; Yoshikawa *et al*., 2002), and our work shows that they have this major impact by concurrently altering several ecosystem characteristics that would otherwise provide shallow active layers. Fire severity ranges widely within boreal forests, and our study suggests that fires of differing intensity will have different levels of influence on ALT, with less severe impacts where only tree and understory canopies are removed compared to fires where insulating moss and OM layers are also lost (Turetsky *et al.,* 2011). It is therefore of considerable concern that both forest fire frequency and intensity are predicted to increase with climate change; indeed clear increases in North American boreal forest affected by fire have been observed over recent years (Gillett *et al*., 2004; Kasischke & Turetsky, 2006). Increased fire intensities will lead to greater ALT increases, and shorter intervals between fires will lessen permafrost recovery, leading to loss of permafrost which may shift black spruce ecosystems from being a C source to a sink (Jorgenson *et al*., 2010; O'Donnell *et al*., 2011). However, in areas where permafrost is a driver of soil conditions, and thin active layers can impede drainage and increase soil moisture contents, the depth of burn may be reduced, as shown in lowlands in Alaska (Turetsky *et al*., 2011). In such ecosystems, fire may have less effect on permafrost‐protecting ecosystem characteristics and thus there may be more potential for ecosystem recovery.

Finally, while the selected sites allow particular insight into how fire drives deepening of ALTs, the relationships seen might also be used to determine the extent that other processes altering vegetation structure (not directly assessed here) may result in deeper ALTs (e.g. stand damaging forest pests and disease, drought, storms; Gauthier *et al*., 2015).

### Permafrost as a responder to, or driver of, ecosystem characteristics

In our study, permafrost is considered to be a responder to ecosystem characteristics, rather than a driver of them. At our study sites, vegetation and soil properties drive permafrost because the permafrost here is climate‐driven and ecosystem‐protected *sensu* Shur & Jorgenson (2007) (Morse *et al*., 2015), and evapotranspiration is the main component of the surface energy balance (Burn, 1998). Hence, vegetation (through evapotranspiration) and summer rainfall determine surface and deeper soil moisture in our study, rather than the presence of permafrost itself. In other areas, especially where permafrost impedes drainage and promotes peat accumulation, permafrost can be a key driver of ecosystem dynamics. This occurs, for example in flat terrain with low‐centred ice‐wedge polygons in Arctic lowlands or where a bowl‐shaped permafrost table drives cryoturbation in nonsorted (e.g. hummocky) patterned ground (Mackay, 1980).

Our approach of conducting detailed surveys across multiple land cover types has allowed us to determine the relative importance of the critical factors controlling ALT in boreal forest, and has also revealed how they interact to modify ALT. Moss layer thickness, tree canopy LAI and organic layer thickness are demonstrated to play critical roles in determining ALTs in boreal forest, which underlines the importance of including these components in processed‐based models, and of testing models that include vegetation‐soil interactions against data sets such as that presented here. Crucially though, the importance of these influences is highly dependent on soil moisture. This result suggests that changes in the magnitude and timing of precipitation, along with changes in evapotranspiration, could dramatically alter the interactions between vegetation, soil and permafrost. The impacts of future changes in precipitation and evapotranspiration on ALT and permafrost degradation require much more attention. We also demonstrate that forest fires influence ALT by simultaneously removing or reducing multiple ecosystem components that would otherwise reduce ALTs and protect permafrost. Again, this raises further concern in light of the already increasing fire frequency and intensity in boreal regions. Understanding the mechanisms through which vegetation and edaphic factors determine ALT and how they will interact with future changes in fire regime or precipitation patterns is vital in order to predict future rates and carbon cycle consequences of permafrost degradation in a changing climate.

## Supporting information


**Data S1.** Regional setting, Justification of SEM design, Discussion of individual site ALTs.
**Fig. S1.** Map showing location of study sites.
**Fig. S2.** Percentage cover of understory species within the four sites.
**Fig. S3.** Growing season (1st June 2014 – 1st September 2014) daily climate data.
**Table S1.** Summary statistics for each of the parameters measured at the four field sites.
**Table S2.** Parameter estimates for a multiple regression models of the effect of the vegetation and edaphic variables on thaw depth within each site.
**Table S3.** SEM parameters.

## References

[gcb13248-bib-0001] Aiken LS , West SG (1991) Multiple Regression: Testing and Interpreting Interactions. Sage Publications, Thousand Oaks, CA, USA.

[gcb13248-bib-0002] Arbuckle JL (2013) IBM SPSS Amos 22 User's Guide. Amos Development Corporation, Crawfordville, FL, USA.

[gcb13248-bib-0003] Baltzer J , Veness T , Chasmer LE , Sniderhan AE , Quinton WL (2014) Forests on thawing permafrost: fragmentation, edge effects, and net forest loss. Global Change Biology, 20, 824–834. 23939809 10.1111/gcb.12349

[gcb13248-bib-0004] Benjamini Y , Hochberg Y (1995) Controlling the false discovery rate: a practical and powerful approach to multiple testing. Journal of the Royal Statistical Society: Series B, 57, 289–300.

[gcb13248-bib-0005] Blok D , Heijmans MMPD , Schaepman‐Strub G , Kononov AV , Maximov TC , Berendse F (2010) Shrub expansion may reduce summer permafrost thaw in Siberian tundra. Global Change Biology, 16, 1296–1305.

[gcb13248-bib-0006] Burn CR (1998) The response (1958–1997) of permafrost and near‐surface ground temperatures to forest fire, Takhini River valley, southern Yukon Territory. Canadian Journal of Earth Sciences, 35, 184–199.

[gcb13248-bib-0007] Burn CR , Smith CAS (1988) Observations of the” thermal offset” in near‐surface mean annual ground temperatures at several sites near Mayo, Yukon Territory, Canada. Arctic, 41, 99–104.

[gcb13248-bib-0008] Canadian Forest Service (2014) Canadian National Fire Database ‐ Agency Fire Data. Natural Resources Canada, Canadian Forest Service, Northern Forestry Centre, Edmonton, Alberta. Available at: http://cwfis.cfs.nrcan.gc.ca/en_CA/nfdb (accessed 15 July 2015).

[gcb13248-bib-0009] Crawley MJ (2012) The R Book. J Wiley & Sons, Chichester, UK.

[gcb13248-bib-0010] Forbes BC , Fauria MM , Zetterberg P (2010) Russian Arctic warming and “greening” are closely tracked by tundra shrub willows. Global Change Biology, 16, 1542–1554.

[gcb13248-bib-0011] Gauthier S , Bernier P , Kuuluvainen T , Shvidenko AZ , Schepaschenko DG (2015) Boreal forest health and global change. Science, 349, 819–822. 26293953 10.1126/science.aaa9092

[gcb13248-bib-0012] Gillett NP , Weaver AJ , Zwiers FW , Flannigan MD (2004) Detecting the effect of climate change on Canadian forest fires. Geophysical Research Letters, 31, L18211.

[gcb13248-bib-0013] Gornall JL , Jónsdóttir IS , Woodin SJ , Van der Wal R (2007) Arctic mosses govern below‐ground environment and ecosystem processes. Oecologia, 153, 931–941. 17618466 10.1007/s00442-007-0785-0

[gcb13248-bib-0014] Grace J (2002) Impacts of climate change on the tree line. Annals of Botany, 90, 537–544. 12324278 10.1093/aob/mcf222 PMC4240388

[gcb13248-bib-0015] Graham MH (2003) Confronting multicollinearity in ecological multiple regression. Ecology, 84, 2809–2815.

[gcb13248-bib-0016] Harden JW , O'Neill KP , Trumbore SE , Veldhuis H , Stocks BJ (1997) Moss and soil contributions to the annual net carbon flux of a maturing boreal forest. Journal of Geophysical Research: Atmospheres, 102, 28805–28816.

[gcb13248-bib-0017] Hinkel KM , Outcalt SI (1994) Identification of heat transfer processes during soil cooling, freezing, and thaw in central Alaska. Permafrost and Periglacial Processes, 5, 217–235.

[gcb13248-bib-0018] Hugelius G , Strauss J , Zubrzycki S *et al* (2014) Estimated stocks of circumpolar permafrost carbon with quantified uncertainty ranges and identified data gaps. Biogeosciences, 11, 6573–6593.

[gcb13248-bib-0019] Iijima Y , Fedorov AN , Park H , Suzuki K , Yabuki H , Maximov TC , Ohata T (2010) Abrupt increases in soil temperatures following increased precipitation in a permafrost region, central Lena River basin, Russia. Permafrost and Periglacial Processes, 21, 30–41.

[gcb13248-bib-0020] IPCC (2013) Long‐term climate change: projections, commitments and irreversibility In: Climate Change 2013: The Physical Science Basis. Contribution of Working Group I to the Fifth Assessment Report of the Intergovernmental Panel on Climate Change (eds Stocker TF, Qin D, Plattner G‐K, Tignor M, Allen SK, Boschung J, Nauels A, Xia Y, Bex V, Midgley PM), pp. 1029–1136. Cambridge University Press, Cambridge, UK.

[gcb13248-bib-0021] Jia GJ , Epstein HE , Walker A (2009) Vegetation greening in the Canadian Arctic related to decadal warming. Journal of Environmental Monitoring, 11, 2231–2238. 20024021 10.1039/b911677j

[gcb13248-bib-0022] Johansson M , Callaghan TV , Bosiö J , Åkerman HJ , Jackowicz‐Korczynski M , Christensen TR (2013) Rapid responses of permafrost and vegetation to experimentally increased snow cover in sub‐arctic Sweden. Environmental Research Letters, 8, 035025.

[gcb13248-bib-0023] Johnson KD , Harden JW , McGuire DA , Clark M , Yuan F , Finley AO (2013) Permafrost and organic layer interactions over a climate gradient in a discontinuous permafrost zone. Environmental Research Letters, 8, 035028.

[gcb13248-bib-0024] Jorgenson MT , Romanovsky V , Harden J *et al* (2010) Resilience and vulnerability of permafrost to climate change. Canadian Journal of Forest Research, 40, 1219–1236.

[gcb13248-bib-0025] Kade A , Walker D (2008) Experimental alteration of vegetation on nonsorted circles: effects on cryogenic activity and implications for climate change in the Arctic. Arctic, Antarctic, and Alpine Research, 41, 119–127.

[gcb13248-bib-0026] Kane DL , Hinkel KM , Goering DJ , Hinzman LD , Outcalt SI (2001) Non‐conductive heat transfer associated with frozen soils. Global and Planetary Change, 29, 275–292.

[gcb13248-bib-0027] Kasischke ES , Turetsky MR (2006) Recent changes in the fire regime across the North American boreal region ‐ Spatial and temporal patterns of burning across Canada and Alaska. Geophysical Research Letters, 33, L09703.

[gcb13248-bib-0028] Kelly R , Chipman ML , Higuera PE , Stefanova I , Brubaker LB , Hu FS (2013) Recent burning of boreal forests exceeds fire regime limits of the past 10,000 years. Proceedings of the National Academy of Sciences of the United States of America, 110, 13055–13060. 23878258 10.1073/pnas.1305069110 PMC3740857

[gcb13248-bib-0029] Lundberg A , Koivusalo H (2003) Estimating winter evaporation in boreal forests with operational snow course data. Hydrological Processes, 17, 1479–1493.

[gcb13248-bib-0030] Mackay JR (1980) The origin of hummocks, western Arctic coast, Canada. Canadian Journal of Earth Sciences, 17, 996–1006.

[gcb13248-bib-0031] Mackay JR (1995) Active layer changes (1968 to 1993) following the forest‐tundra fire near Inuvik, NWT, Canada. Arctic and Alpine Research, 27, 323–336.

[gcb13248-bib-0032] Marsh P , Bartlett P , MacKay M , Pohl S , Lantz T (2010) Snowmelt energetics at a shrub tundra site in the western Canadian Arctic. Hydrological Processes, 24, 3603–3620.

[gcb13248-bib-0033] Morse PD , Wolfe SA , Kokelj SV , Gaanderse AJR (2015) The occurrence and thermal disequilibrium state of permafrost in forest ecotopes of the Great Slave Region, Northwest Territories, Canada. Permafrost and Periglacial Processes, doi: 10.1002/ppp.1858.

[gcb13248-bib-0034] Nelson FE , Hinkel KM (2003) Methods for measuring active‐layer thickness In: A Handbook on Periglacial Field Methods (eds Humlum O, Matsuoka N), pp. 10–20. University of the North in Svalbard, Longyearbyen, Norway.

[gcb13248-bib-0035] Nowinski NS , Taneva L , Trumbore SE , Welker JM (2010) Decomposition of old orgianic matter as a result of deeper active layers in a snow depth manipulation experiment. Oceologia, 163, 785–792. 10.1007/s00442-009-1556-x PMC2886135 20084398

[gcb13248-bib-0036] O'Donnell JA , Romanovsky VE , Harden JW , McGuire AD (2009) The effect of moisture content on the thermal conductivity of moss and organic soil horizons from black spruce ecosystems in Interior Alaska. Soil Science, 174, 646–651.

[gcb13248-bib-0037] O'Donnell JA , Harden JW , McGuire AD , Kanevskiy MZ , Jorgenson MT , Xu X (2011) The effect of fire and permafrost interactions on soil carbon accumulation in an upland black spruce ecosystem of interior Alaska: implications for post‐thaw carbon loss. Global Change Biology, 17, 1461–1474.

[gcb13248-bib-0038] Osterkamp TE , Viereck L , Shur Y , Jorgenson MT , Racine C , Doyle A , Boone RD (2000) Observations of thermokarst and its impact on boreal forests in Alaska, U.S.A. Arctic, Antarctic and Alpine Research, 32, 303–315.

[gcb13248-bib-0039] R Core Team (2014) R: A Language and Environment for Statistical Computing. R Foundation for Statistical Computing, Vienna, Austria Available at: http://www.R-project.org/ (accessed 11 Feb 2015).

[gcb13248-bib-0041] Riseborough DW , Wolfe SA , Duchesne C (2013) Permafrost modelling in northern Slave region Northwest Territories, Phase 1: Climate data evaluation and 1‐d sensitivity analysis; Geological Survey of Canada. Open File, 7333, 50 p.

[gcb13248-bib-0042] Romanovsky VE , Smith SL , Christiansen HH (2010) Permafrost thermal state in the polar Northern Hemisphere during the international polar year 2007‐2009: a synthesis. Permafrost and Periglacial Processes, 21, 106–116.

[gcb13248-bib-0043] Schaefer K , Zhang T , Bruhwiler L , Barrett AP (2011) Amount and timing of permafrost carbon release in response to climate warming. Tellus Series B, 63, 165–180.

[gcb13248-bib-0044] Schuur EAG , Bockheim J , Canadell JG *et al* (2008) Vulnerability of permafrost carbon to climate change: implications for the global carbon cycle. BioScience, 58, 701–714.

[gcb13248-bib-0045] Schuur EAG , Vogel JG , Crummer KG , Lee H , Sickman JO , Osterkamp TE (2009) The effect of permafrost thaw on old carbon release and net carbon exchange from tundra. Nature, 459, 556–559. 19478781 10.1038/nature08031

[gcb13248-bib-0046] Shiklomanov NI , Nelson FE (2013) Active layer processes In: Encyclopedia of Quaternary Sciences, 2nd edn (eds Elias SA, Mock CJ) Vol. 3, pp. 421–429. Elsevier, Amsterdam, The Netherlands.

[gcb13248-bib-0047] Shiklomanov NI , Streletskiy DA , Nelson FE *et al* (2010) Decadal variations of active‐layer thickness in moisture‐controlled landscapes, Barrow, Alaska. Journal of Geophysical Research, 115, G00I04.

[gcb13248-bib-0048] Shur Y , Jorgenson MT (2007) Patterns of permafrost formation and degradation in relation to climate and ecosystems. Permafrost and Periglacial Processes, 18, 7–19.

[gcb13248-bib-0049] Smith SL , Burgess MM , Riseborough D , Nixon FM (2005) Recent trends from Canadian permafrost thermal monitoring network sites. Permafrost and Periglacial Processes, 16, 19–30.

[gcb13248-bib-0050] Smith SL , Wolfe SA , Riseborough DW , Nixon FM (2009) Active‐layer characteristics and summer climatic indices, Mackenzie Valley, Northwest Territories, Canada. Permafrost and Periglacial Processes, 220, 201–220.

[gcb13248-bib-0051] Soudzilovskaia NA , van Bodegom PM , Cornelissen JHC (2013) Dominant bryophyte control over high‐latitude soil temperature fluctuations predicted by heat transfer traits, field moisture regime and laws of thermal insulation. Functional Ecology, 27, 1442–1454.

[gcb13248-bib-0052] Stocks BJ , Fostberg MA , Lynham TJ *et al* (1998) Climate change and forest fire potential in Russian and Canadian boreal forests. Climatic Change, 23, 1–13.

[gcb13248-bib-0053] Street LE , Stoy P , Sommerkorn M , Fletcher BJ , Sloan V , Hill TC , Williams M (2012) Seasonal bryophyte productivity in the sub‐arctic: a comparison with vascular plants. Functional Ecology, 36, 365–378.

[gcb13248-bib-0054] Street LE , Subke JA , Sommerkorn M , Sloan V , Ducrotoy H , Phoenix GK , Williams M (2013) The role of mosses in carbon uptake and partitioning in arctic vegetation. New Phytologist, 199, 163–175. 23614757 10.1111/nph.12285

[gcb13248-bib-0055] Sturm M , McFadden J , Liston G (2001) Snow‐shrub interactions in Arctic tundra: a hypothesis with climatic implications. Journal of Climate, 14, 336–344.

[gcb13248-bib-0056] Tobin J (1958) Estimation of relationships for limited dependent variables. Ecomometrica, 26, 24–36.

[gcb13248-bib-0057] Turetsky MR , Wieder RK , Vitt DH , Evans RJ , Scott KD (2007) The disappearance of relict permafrost in boreal north America: effects on peatland carbon storage and fluxes. Global Change Biology, 13, 1922–1934.

[gcb13248-bib-0058] Turetsky MR , Kane ES , Harden JW , Ottmar RD , Maines KL , Hoy E , Kasischke ES (2011) Recent acceleration of biomass burning and carbon losses in Alaskan forests and peatlands. Nature Geoscience, 4, 27–31.

[gcb13248-bib-0059] Turetsky MR , Bond‐Lamberty B , Euskrichen E , Talbot J , Frolking S , McGuire AD , Tuittila E‐S (2012) The resilience and functional role of moss in boreal and arctic ecosystems. New Phytologist, 196, 49–67. 22924403 10.1111/j.1469-8137.2012.04254.x

[gcb13248-bib-0060] Viereck LA , Werdin‐Pfisterer NR , Adams PC , Yoshikawa K (2008) Effect of wildfire and fireline construction on the annual depth of thaw in a black spruce permafrost forest in interior Alaska: a 36‐year record of recovery In: Proceedings of the Ninth International Conference on Permafrost (eds Kane DL, Hinkel KM) Vol. 29, pp. 1845–1850. University of Alaska Fairbanks, Fairbanks, AK, USA.

[gcb13248-bib-0061] Walker DA , Jia GJ , Epstein HE *et al* (2003) Vegetation‐soil‐thaw‐depth relationships along a low‐arctic bioclimate gradient, Alaska: synthesis of information from the ATLAS studies. Permafrost and perigalacial processes, 14, 103–123.

[gcb13248-bib-0062] Walker MD , Wahren CH , Hollister RD *et al* (2006) Plant community responses to experimental warming across the tundra biome. Proceedings of the National Academy of Sciences of the United States of America, 103, 1342–1346. 16428292 10.1073/pnas.0503198103 PMC1360515

[gcb13248-bib-0063] Weiss M , Baret F (2010) CAN‐EYE v6.1 User Manual. INRA, Paris, France.

[gcb13248-bib-0064] White JD , Running SW , Nemani R , Keane RE , Ryan KC (1997) Measurement and remote sensing of LAI in Rocky Mountain montane ecosystems. Canadian Journal of Forest Research, 27, 1714–1727.

[gcb13248-bib-0065] Yee TW (2014) VGAM: Vector Generalized Linear and Additive Models. R package version 0.9‐5. Available at: http://CRAN.R-project.org/package=VGAM (accessed 23 April 2015).

[gcb13248-bib-0066] Yi S , Woo MK , Arain MA (2007) Impacts of peat and vegetation on permafrost degradation under climate warming. Geophysical Research Letters, 34, L16504.

[gcb13248-bib-0067] Yoshikawa K , Bolton WR , Romanovsky VE , Fukuda M , Hinzman LD (2002) Impacts of wildfire on the permafrost in the boreal forests of Interior Alaska. Journal of Geophysical Research: Atmospheres, 107, FFR‐4.

[gcb13248-bib-0068] Zimov SA , Schuur EAG , Chapin FS (2006) Climate change. Permafrost and the global carbon budget. Science, 312, 1612–1613. 16778046 10.1126/science.1128908

