# Supplementary material for: The influence of vegetation and soil characteristics on active‐layer thickness of permafrost soils in boreal forest
Source: Glob Chang Biol. 2016 Jun 9;22(9):3127–40. doi: 10.1111/gcb.13248 (PMC4999035; doi:10.1111/gcb.13248)
Supplement: Supplementary file 1 — Data S1. Regional setting, Justification of SEM design, Discussion of individual site ALTs. Fig. S1. Map showing location of study sites. Fig. S2. Percentage cover of understory species within the four sites. Fig. S3. Growing season (1st June 2014 – 1st September 2014) daily climate data. Table S1. Summary statistics for each of the parameters measured at the four field sites. Table S2. Parameter estimates for a multiple regression models of the effect of the vegetation and edaphic variables on thaw depth within each site. Table S3. SEM parameters. [file GCB-22-3127-s001.docx]

Supporting information for “*The influence of vegetation and soil characteristics on active-layer thickness of permafrost soils in boreal forest*”

James P. Fisher, Cristian Estop-Aragonés, Aaron Thierry, Dan J. Charman, Steven Wolfe, Iain P. Hartley, Julian B. Murton, Mathew Williams, Gareth K. Phoenix

# Regional Setting

In 2013 we established four field sites near Yellowknife, NWT, Canada (Fig. S1), as representative of permafrost developed in the High Boreal Ecoregion (Ecosystem Classification Group, 2008) of the extensive discontinuous permafrost zone (Heginbottom *et al*., 1995). The Yellowknife region has a continental climate characterised by a mean annual air temperature of –4.3°C (Environment Canada, 2015 – climate normals from 1981 to 2010 for Yellowknife). The mean daily maximum temperature for July is 21.3°C and the mean daily minimum temperature for January is –29.5°C. The mean total annual precipitation is 289 mm, with 41% falling as snow. The snow cover develops in October and persists until late April, peaking at about 38 cm in March.

The region between Yellowknife and Behchoko lies within the Great Slave Lowland of the Taiga Shield High Boreal Ecoregion, which borders the North Arm of Great Slave Lake (Ecosystem Classification Group, 2008). The landscape has developed on the Canadian Shield and is characterised by granitic bedrock outcrops interspersed by rock-floored basins. These basins are partly filled with fine-grained silts and clays of glaciolacustrine and lacustrine origin, with till or glaciofluvial sand and gravel sometimes found beneath them (Kerr and Wilson, 2000). Many basins host peat bogs, fens or lakes. Brunisols are common on glacial deposits, and Cryosols on peat plateaus. Vegetation comprises black spruce (*Picea mariana*) forest with a heath understory, barren bedrock with sparse jackpine (*Pinus banksiana*) and mixed black spruce-deciduous forest, which occupy about 37%, 25% and 25%, respectively, of the land (Morse *et al*., 2015). Paper birch (*Betula papyrifera*) forests with an herbaceous understory and open peatlands comprise about 1% and 2%, respectively, of land cover, and the remaining land areas consist of fens and bogs.

Permafrost in the Great Slave Lowland is warm and discontinuous, with many abrupt transitions from frozen to unfrozen ground over short distances (Wolfe *et al*., 2011), due largely to variations in organic layer thickness (OLT) (Zhang *et a*l., 2014 ). Permafrost is most widespread in fine-grained sediments beneath forest, with smaller areas of permafrost in peatlands (Morse *et al*., 2015). It tends to be absent within bedrock, fens and bogs (Wolfe, 1998). Brown (1973) reported permafrost about 50 m thick beneath spruce peatland supporting black spruce and some tamarack up to 7 m high, and permafrost about 30 m thick beneath sedge peatland supporting scattered and stunted black spruce and tamarack up to 3 m high. Morse *et al*. (2015) reported that mean annual ground temperatures in permafrost range from –1.4°C beneath undisturbed black spruce forest to –0.02°C beneath a burned and cut peatland; depths of zero annual amplitude are commonly less than 7 m on account of high latent heat effects in the active layer and permafrost. Permafrost near Yellowknife is promoted, in part, by a thermal offset of about 1–4°C in the active layer, explaining why the mean annual ground surface temperatures measured in four peatlands exceeded 0°C (Karunaratne *et al*., 2008). Segregated ground ice is abundant in permafrost developed in silt and clay, and accounts for the occurrence of nearly 1800 lithalsas in the lowland, mainly associated with birch forest (Wolfe *et al*., 2014). Permafrost warming and thawing during recent decades have been widespread in the lowland, and process-based modelling by Zhang *et al*. (2014) indicates that the average permafrost probability (permafrost extent) fell from 72% in the 1950s to 52% in the 2000s, and is projected to fall further to 12.4% in the 2050s and to just 2.5% in the 2090s.

West of Behchoko, the landscape and ecoregion change from the taiga shield to the taiga plains. The plains are underlain by Palaeozoic sedimentary rocks on the Interior Platform. In the Great Slave Plain High Boreal Ecoregion (Ecosystem Classification Group, 2009 ), the bedrock is characterised by limestones, dolomites and sandstones that form a landscape of rocky escarpments, marl ponds and wetlands, sinkholes and beach ridges. Water-washed gravelly and calcareous till is common, and lacustrine and aeolian sediments are present. Brunisolic soils of coarse texture are found throughout the Great Slave Plain. White spruce (*Picea glauca*) forest with bearberry (*Arctostaphylos uva-ursi*) and lichen understories is common, with extensive stands of jack pine in areas frequently burned. Fens are typically dominated by sedges. Permafrost conditions are largely unknown, although peat plateaus are thought to be uncommon.

# Justification of SEM design

**Moss layer direct effect** – The dry moss layer contains a large volume of air and hence is expected to reduce downward heat conduction during dry summer conditions, hence reducing ALT compared to that beneath a wet moss layer.

**Moss layer moisture mediated effect** - Water retention increases with increasing bulk density. Because moss has a lower bulk density than soil organic matter (OM) a thicker moss layer would reduce moisture retention and hence would be expected to result in drier soil.

**OM (soil organic matter) direct effect** – Organic matter contains a large volume of air which would reduce downwards heat conduction during dry summer conditions and so would reduce ALT.

**OM moisture mediated effect** – Feathermoss covered OM is typically dry throughout the whole organic layer during summer (Harden *et al.* 1997). With increasing OM thickness the deeper moisture will have be measured at a point further from the wetter mineral soil and hence would be expected to be drier than with a thin OM layer.

**LAI_Tree_ direct effect** – Increasing LAI_Tree_ is expected to reduce ALT by reducing the amount of radiation reaching the ground surface.

**LAI_Tree_ moisture mediated effect** – Transpiration is expected to increase with greater LAI_Tree_, which would reduce soil moisture.

**LAI_U_ direct effect** – Increasing LAI_U_ is expected to reduce ALT by reducing the amount of radiation reaching the ground surface.

**LAI_U_ moisture mediated effect** – Transpiration is expected to increase with greater LAI_U_, which would reduce soil moisture.

**Slope direct effect** – As slope angle increases up to a certain point on hillslopes facing the sun, more solar radiation will be intercepted, resulting in increased ALTs

**Slope moisture mediated effect** – Runoff may be promoted on steeper slopes which would decrease volumetric soil moisture content.

**Vegetation height** – Taller, shrubby vegetation may be more likely to trap snow causing a thicker snowpack which would insulate the ground in winter and result in an increased ALT. Vegetation height is not expected to have any moisture mediated effects.

**Deeper moisture direct effect** – Increased soil moisture would increase soil thermal conductivity and so is expected to increase ALT.

**Deeper moisture effect on surface moisture** – Near-surface moisture is expected to buffer the moisture content of the surface layer, with moister deeper-soil conditions maintaining a higher volumetric moisture content in the surface layer.

**Surface moisture direct effect** - Increased soil moisture would increase soil thermal conductivity and so would be expected to increase ALT.

# Individual site ALTs

While our main approach focusses on seeking emergent drivers of ALT that operate across land-cover types, our work also provides insight into the key differences in drivers of ALT between the sites and within each land cover type, despite the inevitable weaker statistical power of the experimental design for this individual site analysis.

The birch site had an intermediate ALT compared to the other three sites. It also had a relatively high tree canopy LAI, and this was the only factor that proved to be significant in this land-cover-type’s analysis (Table S2). Smaller ALTs where the birch tree canopy LAI was larger seems most likely due to shading in summer, as these deciduous trees have a poor capacity for the canopy snow trapping mechanism that keeps snow off the ground and ground temperatures cool. This site also lacked a moss layer, instead having a layer of leaf litter which tended to retain more moisture at the surface than the moss in the two black spruce sites. This would have acted to increase thaw depth by increasing downward heat conduction in summer as revealed by our cross-site analysis.

The black spruce sites (MSU and BS) had the shallowest active layer depths of all four sites. The land-cover-type analysis for black spruce (combining these two sites, Table S2) showed increasing OM thickness and increasing tree canopy LAI to be significant drivers in reducing ALT in black spruce forest, but that increasing surface moisture was associated with increasing thaw depth. The two black spruce sites had very similar ALTs in spite of more than 60 km of separation. This appeared to be because the two sites had slightly different combinations of the factors which favour a small ALT. MSU had a smaller tree canopy LAI than BS, which could have led to a larger ALT, but this was compensated for by thicker moss and organic layers at MSU, both of which reduce ALT.

The burned spruce site (MSB) had the greatest mean ALT of all our sites, consistent with it having the wettest soil, a very thin moss layer, and the thinnest OM layer – all factors promoting a deeper ALT. Indeed, with most of the other factors that control ALT reduced or removed by fire (OM thickness, moss depth, LAI_Tree_), only LAIU remains as the main controlling factor in ALT across the burned site (Table S2). Boreal forest fires are known to have a dramatic impact on active layer depth (Yoshikawa *et al*. 2002), and our work shows that they have this major impact by concurrently altering a number of ecosystem characteristics that would otherwise provide shallow active layers. Fire severity can range widely within boreal forests and our study suggests that fires of differing intensity will have different levels of impact on ALT. For fires in which only the canopy or understorey vegetation is burned, we would predict increases in ALT due to both the loss of shading by the reduced tree and understorey LAI and the increases in soil moisture caused by reduced evapotranspiration. However, in more severe fires where more leaf area is consumed as well as the moss and organic layers, we would predict much larger increases in ALT (Turetsky *et al*. 2010). It is therefore of considerable concern that forest fire frequency and intensity are predicted to increase with climate change, and that clear increases in North American boreal forest affected by fire has been observed over recent years (Gillett *et a*l. 2004, Kasischke and Turetsky 2006). With greater deepening of active layers, and less recovery time between fires, this suggests a far greater potential to not only increase permafrost loss by deepening ALTs, but also less chance of permafrost recovery due to shorter fire-return intervals and recovery occurring in a warmer climate (Jorgenson et al., 2010). Modelling has shown that if the fire regime becomes more frequent and fires become more severe black spruce ecosystems may shift from being a C sink to a source, largely as a result of C losses from deeper organic soil horizons (O’ Donnell et al., 2011).

# Supplementary Figures

**
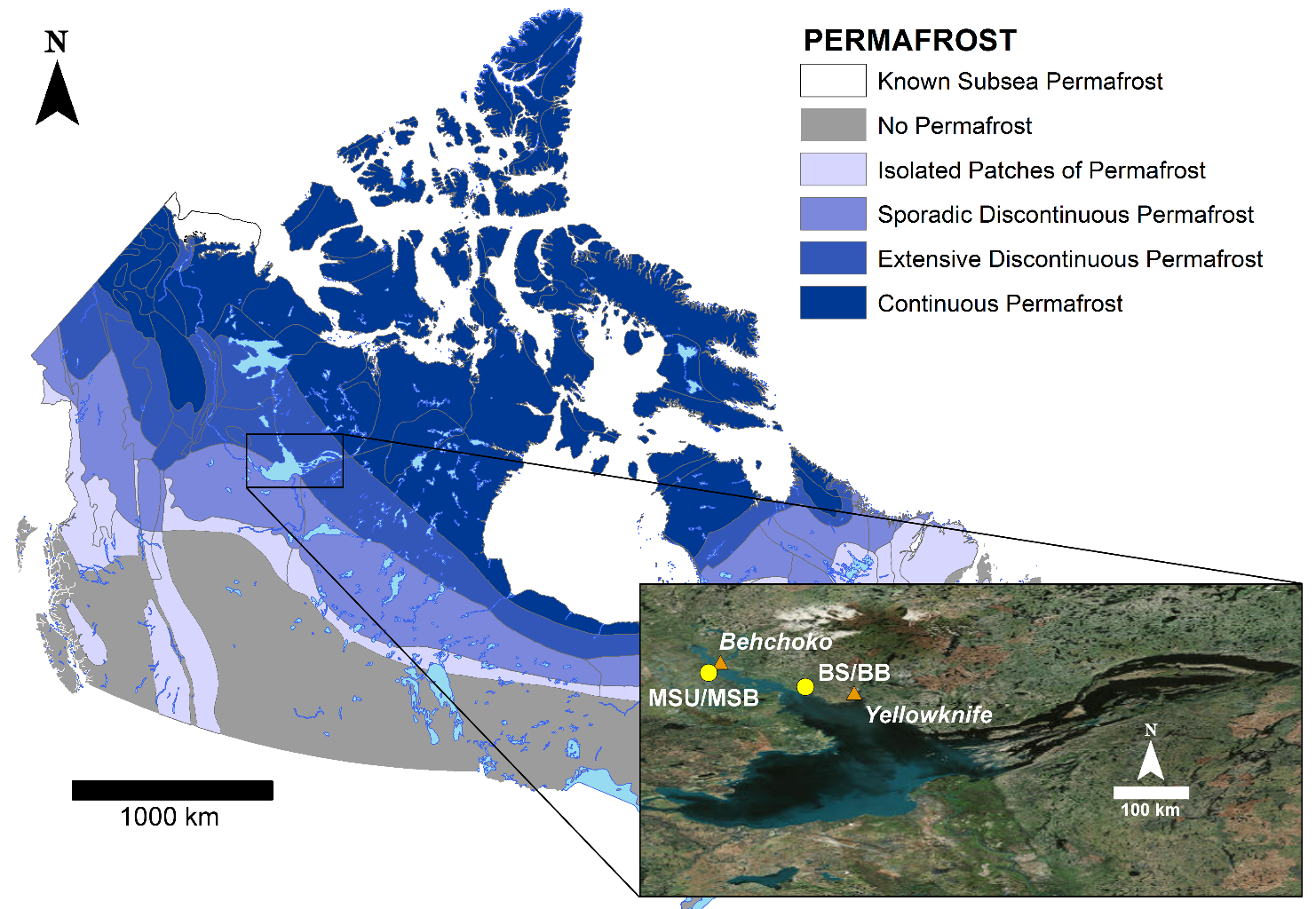
**

**Figure S1.** Map showing location of study sites. MSU is Mosquito Spruce Unburned, MSB Mosquito Spruce Burned, BS is Boundary Creek Spruce and BB is Boundary Creek Birch. Permafrost distribution from Heginbottom *et al.,* 1995.


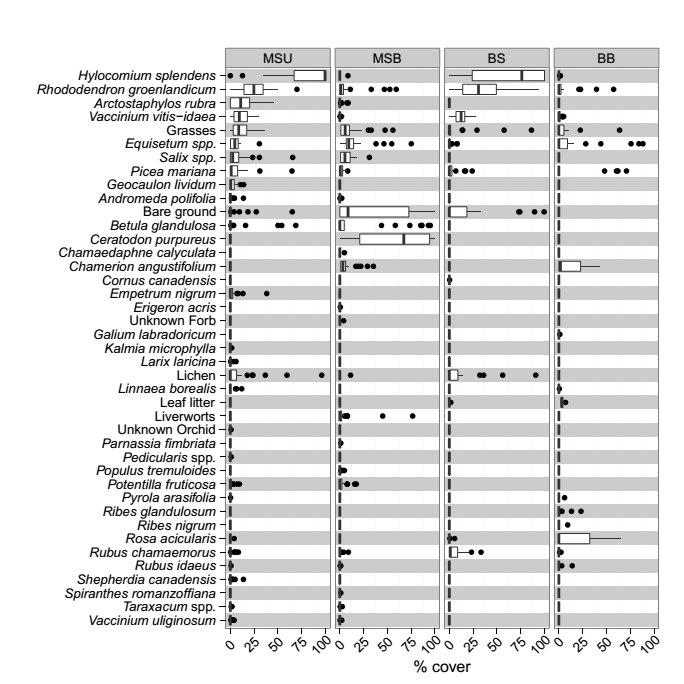


**Figure S2.** Percentage cover of understory species within the four sites. **MSU** is Mosquito Spruce Unburned, **MSB** Mosquito Spruce Burned, **BS** is Boundary Creek Spruce and **BB** is Boundary Creek Birch. Boxplots represent median, 1^st^ and 3^rd^ quartiles (line and box), whiskers represent maximum and minimum and points represent outliers.


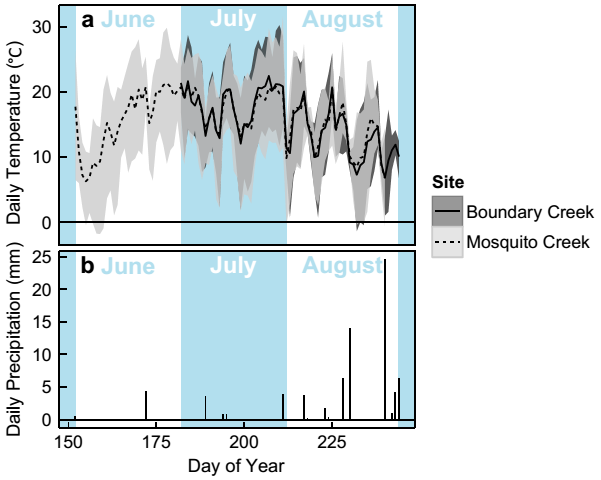


**Figure S3.** Growing season (1st June 2014 – 1st September 2014) daily climate; a) Daily mean (lines) and extreme (shaded area) screened air temperatures at 1 m above ground at the Mosquito Creek and Boundary Creek sites; b) daily precipitation measured at the Environment Canada Yellowknife-Henderson station (62°27'00.0"N, 114°22'48.0"W).

# Supplementary Tables

**Table S1.** Summary statistics for each of the parameters measured at the four field sites. BB, BS, MSB and MSU are the Boundary Creek Birch site, the Boundary Creek Spruce site, the Mosquito Spruce Burned site and the Mosquito Spruce Unburned site, respectively.

| Parameter | Site | Min | 1st Quartile | Median | Mean | 3rd Quartile | Max | SE |
| --- | --- | --- | --- | --- | --- | --- | --- | --- |
| Thaw depth | BB | 43 | 53.3 | 62 | 63.8 | 72.8 | 91 | 2.7 |
| (cm) | BS | 27 | 36.6 | 40.5 | 43.4 | 46.3 | 74.5 | 2.3 |
|  | MSB | 50.5 | 95.2 | 149.4 | 122.7 | 149.6 | 149.8 | 6.16 |
|  | MSU | 8 | 37.1 | 41.5 | 43.4 | 45.8 | 71.5 | 2.2 |
| Organic | BB | 22 | 41 | 56.5 | 57.6 | 66.3 | 106 | 4.7 |
| Matter depth | BS | 21 | 39.5 | 52 | 55.7 | 73.5 | 101 | 4.8 |
| (cm) | MSB | 15 | 34.3 | 43 | 46.3 | 59.8 | 80 | 3.1 |
|  | MSU | 33 | 52 | 64 | 64.1 | 76.8 | 93 | 3 |
| Moss depth | BB | 0 | 0 | 0 | 0 | 0 | 0 | 0 |
| (cm) | BS | 0 | 2 | 3.3 | 4.7 | 7.3 | 12.5 | 0.7 |
|  | MSB | 0 | 0.3 | 0.4 | 0.6 | 0.6 | 5.5 | 0.2 |
|  | MSU | 0 | 4 | 6 | 7.4 | 8.4 | 33 | 1.2 |
| Tree canopy | BB | 0.97 | 1.378 | 1.46 | 1.49 | 1.575 | 2.15 | 0.05 |
| LAI | BS | 0.63 | 1.168 | 1.54 | 1.452 | 1.702 | 2.04 | 0.08 |
| (m^2^ m^-2^) | MSB | 0 | 0.035 | 0.095 | 0.101 | 0.138 | 0.3 | 0.01 |
|  | MSU | 0.03 | 0.6 | 0.67 | 0.676 | 0.79 | 1.1 | 0.03 |
| Understory | BB | 0.416 | 0.855 | 1.01 | 1.169 | 1.523 | 2.41 | 0.1 |
| LAI | BS | 0.240 | 0.635 | 0.743 | 0.917 | 1.355 | 1.644 | 0.09 |
| (m^2^ m^-2^) | MSB | 0.240 | 0.472 | 0.763 | 0.842 | 1.17 | 1.772 | 0.08 |
|  | MSU | 0.328 | 0.679 | 0.835 | 1.021 | 1.295 | 2.155 | 0.09 |
| Slope | BB | 0.9 | 4.7 | 6.4 | 6.2 | 8.0 | 10.9 | 0.5 |
| (°) | BS | 1.1 | 3.4 | 6.4 | 6.8 | 8.8 | 18.6 | 0.9 |
|  | MSB | 0.9 | 4.7 | 6.1 | 6.2 | 7.0 | 12.8 | 0.5 |
|  | MSU | 1.6 | 5.9 | 7.7 | 8.6 | 11.2 | 18.4 | 0.8 |
| Understory | BB | 10.2 | 21.3 | 30.3 | 37.9 | 51.7 | 80 | 4.1 |
| height | BS | 0 | 15 | 21.3 | 24.1 | 34.1 | 50.4 | 3.1 |
| (cm) | MSB | 2.6 | 13.2 | 27.9 | 36.6 | 49.2 | 122 | 6 |
|  | MSU | 2.4 | 21.3 | 32.3 | 35.4 | 41.9 | 116.4 | 4.4 |
| Deeper | BB | 0.088 | 0.146 | 0.175 | 0.179 | 0.201 | 0.295 | 0.01 |
| moisture | BS | 0.099 | 0.189 | 0.228 | 0.264 | 0.297 | 0.612 | 0.03 |
| (m^3^ m^-3^) | MSB | 0.309 | 0.495 | 0.669 | 0.615 | 0.730 | 0.932 | 0.03 |
|  | MSU | 0.078 | 0.145 | 0.206 | 0.226 | 0.249 | 0.512 | 0.02 |
| Surface | BB | 0.097 | 0.124 | 0.142 | 0.143 | 0.160 | 0.196 | 0.01 |
| moisture | BS | 0.028 | 0.075 | 0.101 | 0.101 | 0.127 | 0.228 | 0.01 |
| (m^3^ m^-3^) | MSB | 0.072 | 0.139 | 0.169 | 0.194 | 0.235 | 0.447 | 0.02 |
|  | MSU | 0.012 | 0.064 | 0.0862 | 0.0834 | 0.106 | 0.160 | 0.01 |

**Table S2**. Parameter estimates for multiple regression models of the effect of the vegetation and edaphic variables on active layer thickness for the black spruce (Mosquito Spruce Unburned and Boundary Creek Spruce combined) and paper birch (Boundary Creek Birch) land cover types, and tobit regression model for the burned black spruce site (Mosquito Burned Spruce). Parameters in bold are significant at α = 0.05.

|  |  | Parameter estimate | SE | *t* | *p* |
| --- | --- | --- | --- | --- | --- |
| Black | **(Intercept)** | **3.908** | **0.038** | **102.217** | **< 0.001** |
| spruce | **Organic matter thickness** | **-0.004** | **0.002** | **-2.351** | **0.023** |
|  | Mean vegetation height | 0.001 | 0.002 | 0.833 | 0.409 |
|  | Understory LAI | -0.061 | 0.076 | -0.803 | 0.426 |
|  | **Tree canopy LAI** | **-0.13** | **0.064** | **-2.027** | **0.049** |
|  | Slope | -0.003 | 0.007 | -0.466 | 0.643 |
|  | Deeper moisture | 0.022 | 0.277 | 0.078 | 0.938 |
|  | **Surface moisture** | **2.908** | **0.782** | **3.719** | **< 0.001** |
| Paper | **(Intercept)** | **4.327** | **0.103** | **41.941** | **< 0.001** |
| birch | Mean understory height | 0.003 | 0.002 | 1.374 | 0.184 |
|  | **Tree canopy LAI** | **-0.326** | **0.153** | **-2.136** | **0.045** |
| Burned | **(Intercept)** | **6.531** | **1.312** | **4.977** | **< 0.001** |
| black | **Understory LAI** | **-0.712** | **0.268** | **-2.655** | **0.008** |
| spruce | Tree canopy LAI | 2.247 | 1.731 | 1.298 | 0.194 |
|  | Surface moisture | 2.105 | 1.517 | 1.388 | 0.165 |

**Table S3.** Unstandardized parameter estimates for SEM of the effect of the vegetation and edaphic variables on active layer thickness across all sites. Mean is posterior mean, SE reports distance of Monte-Carlo posterior mean from true posterior mean, SD is likely distance of true unknown parameter from the posterior mean, 95% lower and 95% upper report boundaries of 95% HDI. See Fig. 5 in main text for diagrammatic representation of path coefficients.

|  | Mean | SE | SD | 95% Lower | 95% Upper | Skewness |
| --- | --- | --- | --- | --- | --- | --- |
| **Path coefficients** |  |  |  |  |  |  |
| ALT🡨 Moss Thickness | -2.291 | 0.023 | 0.681 | -3.585 | -0.957 | 0.087 |
| ALT🡨 OM thickness | -0.371 | 0.005 | 0.136 | -0.630 | -0.097 | 0.071 |
| Surface moisture 🡨 Moss thickness | -0.005 | 0.000 | 0.001 | -0.008 | -0.003 | -0.080 |
| Deeper moisture 🡨Moss thickness | -0.011 | 0.000 | 0.003 | -0.017 | -0.005 | -0.015 |
| ALT🡨 Surface moisture | 134.636 | 2.701 | 61.074 | 17.422 | 257.865 | 0.155 |
| ALT🡨 Deeper moisture | 63.755 | 0.660 | 20.074 | 24.243 | 102.822 | -0.050 |
| Deeper moisture 🡨 OM thickness | -0.002 | 0.000 | 0.001 | -0.003 | -0.001 | 0.058 |
| Surface moisture 🡨 Deeper moisture | 0.116 | 0.001 | 0.035 | 0.048 | 0.186 | 0.120 |
| ALT 🡨 Vegetation Height | 0.008 | 0.005 | 0.142 | -0.275 | 0.287 | 0.001 |
| ALT🡨 Slope | 0.527 | 0.041 | 0.824 | -1.103 | 2.105 | -0.094 |
| Surface moisture 🡨 Slope | -0.003 | 0.000 | 0.002 | -0.006 | 0.001 | 0.084 |
| Deeper moisture 🡨 Slope | -0.010 | 0.000 | 0.004 | -0.018 | -0.001 | 0.078 |
| ALT 🡨 LAI_Tree_ | -21.692 | 0.238 | 5.862 | -33.420 | -10.639 | -0.095 |
| ALT 🡨 LAI_U_ | -5.660 | 0.262 | 7.475 | -20.754 | 9.071 | -0.009 |
| Deeper moisture 🡨 LAI_Tree_ | -0.210 | 0.001 | 0.022 | -0.255 | -0.166 | -0.069 |
| Surface moisture 🡨 LAI_Tree_ | -0.011 | 0.000 | 0.011 | -0.033 | 0.011 | -0.003 |
| Surface moisture🡨 LAI_U_ | 0.024 | 0.000 | 0.011 | 0.003 | 0.046 | 0.049 |
| Deeper moisture 🡨 LAI_U_ | -0.060 | 0.001 | 0.029 | -0.116 | -0.001 | 0.006 |
| Surface moisture 🡨 OM thickness | 0.000 | 0.000 | 0.000 | -0.001 | 0.000 | 0.152 |
| **Means** |  |  |  |  |  |  |
| Moss thickness | 3.228 | 0.021 | 0.493 | 2.276 | 4.184 | -0.072 |
| OM thickness | 52.726 | 0.058 | 2.053 | 48.717 | 56.803 | 0.001 |
| LAI_U_ | 0.981 | 0.002 | 0.050 | 0.882 | 1.079 | 0.002 |
| LAI_Tree_ | 0.867 | 0.003 | 0.065 | 0.735 | 0.997 | -0.049 |
| Slope | 6.972 | 0.012 | 0.360 | 6.267 | 7.676 | 0.026 |
| Vegetation Height | 33.790 | 0.083 | 2.566 | 28.819 | 38.963 | 0.074 |
| **Intercepts** |  |  |  |  |  |  |
| ALT | 81.369 | 0.816 | 18.953 | 44.134 | 118.063 | -0.025 |
| Surface moisture | 0.119 | 0.001 | 0.035 | 0.049 | 0.185 | -0.132 |
| Deeper moisture | 0.786 | 0.003 | 0.058 | 0.673 | 0.900 | 0.034 |
| **Covariances** |  |  |  |  |  |  |
| Moss thickness ↔ OM thickness | 3.543 | 0.577 | 11.078 | -18.523 | 26.072 | -0.041 |
| Moss thickness ↔ LAI_Tree_ | 0.078 | 0.021 | 0.357 | -0.642 | 0.794 | -0.007 |
| OM thickness ↔ LAI_Tree_ | 0.737 | 0.054 | 1.509 | -2.129 | 3.977 | 0.284 |
| OM thickness ↔ LAI_U_ | -0.076 | 0.044 | 1.069 | -2.200 | 2.093 | 0.102 |
| Vegetation Height ↔ OM thickness | -36.80 | 2.985 | 57.195 | -146.92 | 78.277 | 0.042 |
| OM thickness ↔ Slope | 5.211 | 0.408 | 7.852 | -10.337 | 20.645 | -0.027 |
| Moss thickness ↔ LAI_U_ | 0.369 | 0.015 | 0.291 | -0.175 | 0.967 | 0.224 |
| Vegetation Height ↔ Moss thickness | 12.241 | 0.810 | 14.890 | -14.654 | 44.279 | 0.347 |
| Moss thickness ↔ Slope | 6.759 | 0.097 | 2.135 | 2.869 | 11.339 | 0.368 |
| LAI_Tree_ ↔ LAI_U_ | 0.049 | 0.002 | 0.036 | -0.020 | 0.122 | 0.104 |
| Vegetation Height ↔ LAI_Tree_ | -1.996 | 0.080 | 1.794 | -5.694 | 1.380 | -0.132 |
| Slope ↔ LAI_Tree_ | -0.119 | 0.012 | 0.258 | -0.640 | 0.369 | -0.188 |
| Vegetation Height ↔ LAI_Tree_ | 8.914 | 0.076 | 1.649 | 5.982 | 12.399 | 0.361 |
| Slope ↔ LAI_U_ | 0.246 | 0.010 | 0.202 | -0.152 | 0.653 | 0.036 |
| Vegetation Height ↔ Slope | 13.367 | 0.327 | 10.768 | -7.683 | 35.503 | 0.044 |
| **Variances** |  |  |  |  |  |  |
| Moss thickness | 26.790 | 0.213 | 3.944 | 19.991 | 35.309 | 0.436 |
| OM thickness | 452.147 | 3.475 | 64.920 | 340.175 | 602.870 | 0.663 |
| Slope | 14.266 | 0.107 | 2.057 | 10.758 | 18.840 | 0.473 |
| LAI_Tree_ | 0.451 | 0.003 | 0.070 | 0.335 | 0.613 | 0.647 |
| LAI_U_ | 0.265 | 0.001 | 0.038 | 0.199 | 0.345 | 0.375 |
| Deeper moisture | 0.022 | 0.000 | 0.003 | 0.016 | 0.029 | 0.551 |
| Surface moisture | 0.003 | 0.000 | 0.000 | 0.002 | 0.004 | 0.432 |
| Vegetation Height | 710.10 | 6.173 | 103.07 | 533.398 | 936.560 | 0.498 |
| ALT | 715.61 | 4.915 | 118.89 | 513.951 | 975.971 | 0.545 |

# References

Brown RJE (1973) Influence of climatic and terrain factors on ground temperatures at three locations in the permafrost region of Canada. In *Proceedings of the Second International Conference on Permafrost, North American Contribution, 13 – 28 July 1973, Yakutsk, U.S.S.R*. National Academy of Sciences, Washington, D.C. pp27-34.

Ecosystem Classification Group (2008) Ecological regions for the Northwest Territories – Taiga Shield. Government of the Northwest Territories, Yellowknife, Canada, 149 pp

Ecosystem Classification Group (2009) Ecological regions for the Northwest Territories – Taiga Plains. Government of the Northwest Territories, Yellowknife, Canada, 173 pp.

Environment Canada (2015) Climate normals and averages 1981–2010. Online data at http://climate.weather.gc.ca/climate_normals/results_1981_2010_e.html.

Gillett NP, Weaver AJ, Zwiers FW, Flannigan MD (2004) Detecting the effect of climate change on Canadian forest fires. Geophysical Research Letters, **31**, L18211

Harden JW, O'Neill KP, Trumbore SE, Veldhuis H, Stocks BJ (1997) Moss and soil contributions to the annual net carbon flux of a maturing boreal forest. Journal of Geophysical Research: Atmospheres, **102**, 28805-28816.

Heginbottom, J.A., Dubreuil, M.A. and Harker, P.A (1995) Canada-Permafrost. In *National Atlas of Canada, 5th Edition*. National Atlas Information Service, Natural Resources Canada, Ottawa, ON, Canada, Plate 2.1. MCR 4177.

Jorgenson MT, Romanovsky V, Harden J, Shur Y, O’Donnell J, Schuur EAG, Kanevskiy M, Marchenko S (2010) Resilience and vulnerability of permafrost to climate change. Canadian Journal of Forest Research, **40**, 1219–1236.

Karunaratne KC, Kokelj SV, Burn CR (2008) Near-surface permafrost conditions near Yellowknife, Northwest Territories, Canada. In *Proceedings, Ninth International Conference on Permafrost, 29 June–3 July 2008, Fairbanks, Alaska* (eds Kane DL, Hinkel KM) pp. 907-912, Institute of Northern Engineering, University of Alaska – Fairbanks, Fairbanks, USA.

Kasischke ES, Turetsky MR (2006) Recent changes in the fire regime across the North American boreal region - Spatial and temporal patterns of burning across Canada and Alaska. Geophysical Research Letters, **33**, L09703

Kerr DE, Wilson P (2000) Preliminary surficial geology studies and mineral exploration considerations in the Yellowknife area, Northwest Territories. Geological Survey of Canada, Current Research 2000-C3, 8 p.

Morse PD, Wolfe SA, Kokelj SV, Gaanderse, AJR (2015) The occurrence and thermal disequilibrium state of permafrost in forest ecotopes of the Great Slave Region, Northwest Territories, Canada. Permafrost and Periglacial Processes, DOI: 10.1002/ppp.1858.

O'Donnell JA, Harden JW, McGuire AD, Kanevskiy MZ, Jorgenson MT, & Xu X (2011) The effect of fire and permafrost interactions on soil carbon accumulation in an upland black spruce ecosystem of interior Alaska: implications for post‐thaw carbon loss. Global Change Biology, **17**, 1461-1474.

Turetsky MR, Kane ES, Harden JW, Ottmar RD, Maines KL, Hoy E, Kasischke ES (2010) Recent acceleration of biomass burning and carbon losses in Alaskan forests and peatlands. Nature Geoscience, **4**, 27-31.

Wolfe SA (1998) Living with Frozen Ground: a field guide to permafrost in Yellowknife, Northwest Territories. Geological Survey of Cananda

Wolfe SA, Duchesne C, Gaanderse A, Houben A, D'Onofrio R, Kokelj SK, and Stevens CW (2011) Report on 2010-11 Permafrost Investigations in the Yellowknife Area, Northwest Territories. Geological Survey of Canada, Open File 6983 and NWT Open Report 2011-009.

Wolfe SA, Stevens CW, Gaanderse AJ, Oldenborger G (2014) Lithalsa distribution, morphology and landscape associations in the Great Slave Lowland, Northwest Territories, Canada. Geomorphology, **204**, 302-313.

Yoshikawa K, Bolton WR, Romanovsky VE, Fukuda M, Hinzman LD (2002) Impacts of wildfire on the permafrost in the boreal forests of Interior Alaska. Journal of Geophysical Research: Atmospheres, **107**, FFR-4.

Zhang Y, Olthof I, Fraser R, Wolfe SA (2014) A new approach to mapping permafrost and change incorporating uncertainties in ground conditions and climate projections. The Cryosphere Discussions, **8**, 1895-1935. DOI: 10.5194/tcd-8-1895-2014.
